# Nanocellulose-Based Scaffolds for Chondrogenic Differentiation and Expansion

**DOI:** 10.3389/fbioe.2021.736213

**Published:** 2021-08-17

**Authors:** Marcin Szustak, Edyta Gendaszewska-Darmach

**Affiliations:** Faculty of Biotechnology and Food Sciences, Institute of Molecular and Industrial Biotechnology, Lodz University of Technology, Lodz, Poland

**Keywords:** cartilage, tissue engineering, nanocellulose, chondrocytes, stem cells, differentiation

## Abstract

Nanocellulose deserves special attention among the large group of biocompatible biomaterials. It exhibits good mechanical properties, which qualifies it for potential use as a scaffold imitating cartilage. However, the reconstruction of cartilage is a big challenge due to this tissue's limited regenerative capacity resulting from its lack of vascularization, innervations, and sparsely distributed chondrocytes. This feature restricts the infiltration of progenitor cells into damaged sites. Unfortunately, differentiated chondrocytes are challenging to obtain, and mesenchymal stem cells have become an alternative approach to promote chondrogenesis. Importantly, nanocellulose scaffolds induce the differentiation of stem cells into chondrocyte phenotypes. In this review, we present the recent progress of nanocellulose-based scaffolds promoting the development of cartilage tissue, especially within the emphasis on chondrogenic differentiation and expansion.

## Introduction

Despite possessing remarkable mechanical properties, articular cartilage has very limited regeneration capacity resulting from the lack of vascularization, lymphangion, innervations, and restricted infiltration of local progenitor cells. Moreover, the cartilage tissue is sparsely populated with chondrocytes embedded within an extracellular matrix (ECM) (Castañeda and Vicente, 2017). Therefore, articular cartilage injuries initiated both traumatically and with systemic diseases and aging, frequently progress to osteoarthritis (OA). In developing countries, osteoarthritis, characterized by gradual loss of articular cartilage, osteophyte formation, and other abnormalities, is a leading cause of chronic pain and disability. Currently, 250 million people are affected by OA due to the combined effects of increases in life expectancy, body mass index, and joint injuries (Hunter and Bierma-Zeinstra, 2019).

**GRAPHICAL ABSTRACT:**
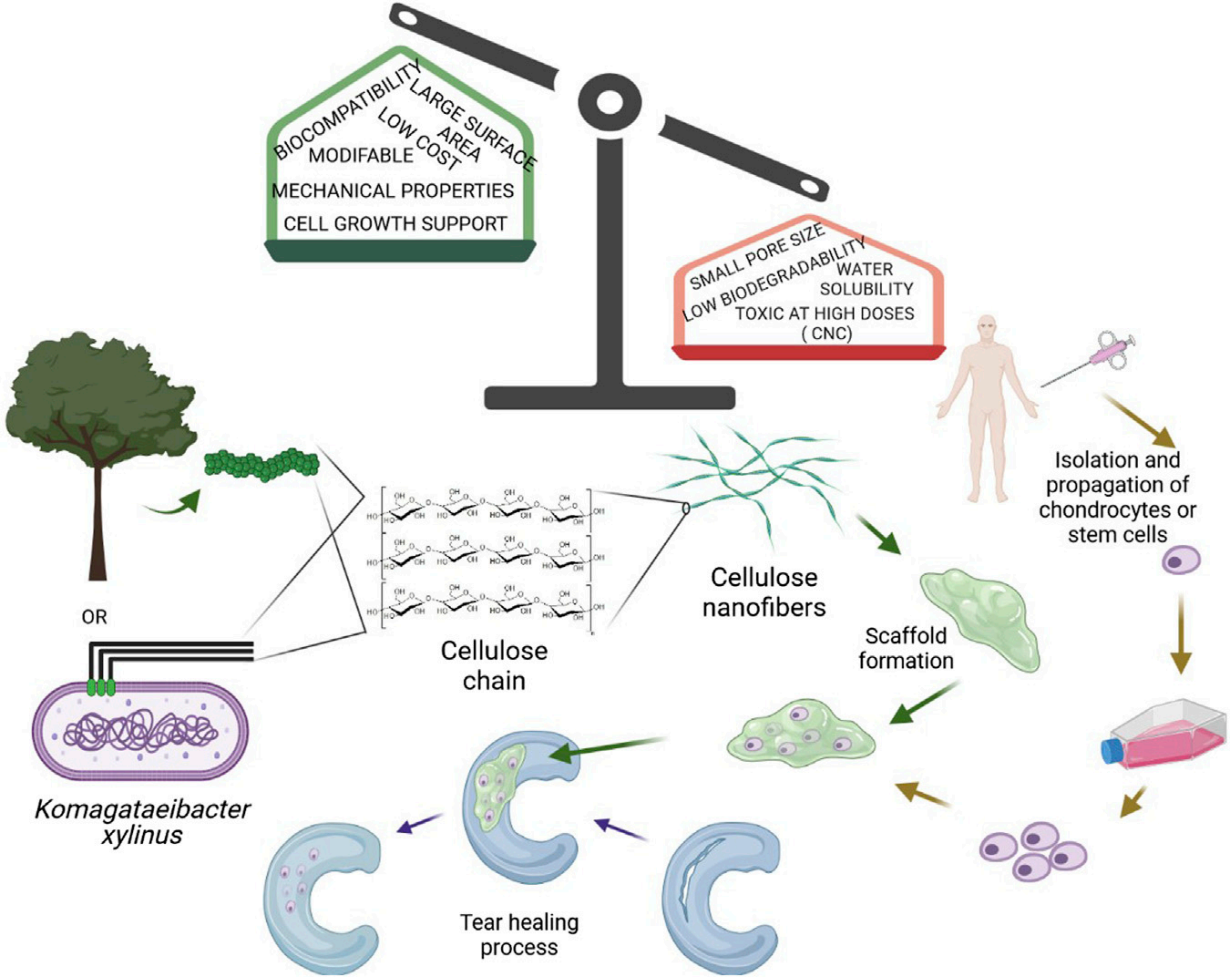


The available treatment options for articular cartilage damage include pharmacological intervention, primarily used for pain management and reducing stiffness, and surgical approaches to treat more advanced stages of cartilage injuries. However, common surgical interventions such as chondroplasty, microfracture, or drilling are effective only for minor defects and provide the relatively short-term functional improvement of joint mobility and reducing pain (Davies and Kuiper, 2019). Restoration of severe cartilage injuries is based on osteochondral autograft or allograft and autologous chondrocyte implantation (ACI). ACI was a breakthrough in the treatment of large articular cartilage defects. In this two-step procedure, chondrocytes are isolated from healthy cartilage (bioptate) and collected arthroscopically, expanded *in vitro* for 2–3 weeks as a monolayer, and embedded into the patient’s damaged tissue with periosteum (Zylińska et al., 2018). Long-term results for first-generation ACI were generally poor, with no significant difference in comparison with microfracture. 20-years follow-up studies showed ACI failure in 37% of treated patients (Ogura et al., 2017). Notably, the critical limitations of ACI is dedifferentiation, defined as the loss of the chondrocyte phenotypes and the adaption of the fibroblast phenotypes during *in vitro* expansion (Mao et al., 2018). Results improved with second-generation techniques, in which a type I-III collagen membrane was used to cover the autologous chondrocytes, and third-generation scaffold-based ACI techniques termed MACI (matrix-induced autologous chondrocyte implantation) (Zylińska et al., 2018).

Following the promising clinical results of MACI, supported by advances in material and biomedical sciences, many unique compositions of scaffolds in cartilage tissue engineering have been proposed (Ngadimin et al., 2021; Zhao et al., 2021). Among natural biomaterials, particular attention has been given to nanocellulose (NC), regarded as “generally recognized as safe” by the U.S. Food and Drug Administration (Halib et al., 2017). Nanocellulose, as the main constituent of plant cell walls, is the most abundant biopolymer in nature. However, it is also synthesized by marine animals, algae, fungi, and several bacteria species (Halib et al., 2017). The most efficient bacterial cellulose producers are acetic acid bacteria of the *Komagataeibacter* genus (Ryngajłło et al., 2020). NC is promising due to its unique biomechanical and rheological properties, such as extraordinarily high stiffness and strength (Piras et al., 2017). Being also biocompatible, insoluble, elastic, and hydrophilic, nanocellulosic materials are of great interest in medical applications such as wound healing or reconstructive surgery of soft and hard tissues (Ludwicka et al., 2019). Numerous reviews have already been published on potential applications of nanocellulose in tissue engineering (Halib et al., 2017; Dutta et al., 2019; Gorgieva and Trček, 2019; Chinta et al., 2021), however, this review will firstly discuss the current state of knowledge in terms of nanocellulose and its composites as scaffolds for chondrogenic expansion and differentiation.

## Tissue Engineering in Cartilage Regeneration–Main Assumptions

In 1993 Langer and Vacanti defined tissue engineering (TE) as “a modern and interdisciplinary science that applies both the principles of engineering and the processes and phenomena of the life sciences toward the development of biological substitutes that restore, maintain, or improve tissue function” (Langer and Vacanti, 1993). Therefore, TE combines biology, clinical medicine, and materials science to produce artificial tissues or organs. In general, regenerative engineering aims to provide a temporary three-dimensional (3D) environment or “scaffold” for recruited or seeded cells to regenerate injured tissues. The tissue engineering triad consists of three critical components that work together to produce a successful construct. The first one, a biomaterial scaffold that temporarily imitates ECM, provides a 3D environment for cell attachment, proliferation, and differentiation. Numerous scaffold properties, such as architecture (*e.g.*, pore size and shape), modulus, chemical functionality, hydrophobicity, and others, have been shown to affect cell phenotype and activity. Scaffolds with high porosity and pore interconnectivity are easily penetrated by cells and help diffusion of nutrients and gases. A relevant collection of cells (stem cells, differentiated cells) serves as the second element while appropriate signals such as chemical mediators (vitamins, amino acids, hormones, growth factors, cytokines, and active drugs), mechanical (compression or pressure), and physicochemical factors (oxidative stress, carbon dioxide concentration, *etc*.) are the third component which affects cell viability and growth (James and Laurencin, 2014; Oprea and Voicu, 2020). A novel material-based regenerative approach relies solely on the chemical and physical properties of the scaffold to guide tissue regeneration (i.e., without exogenous growth factors) (Almouemen et al., 2019).

**FIGURE 1:**
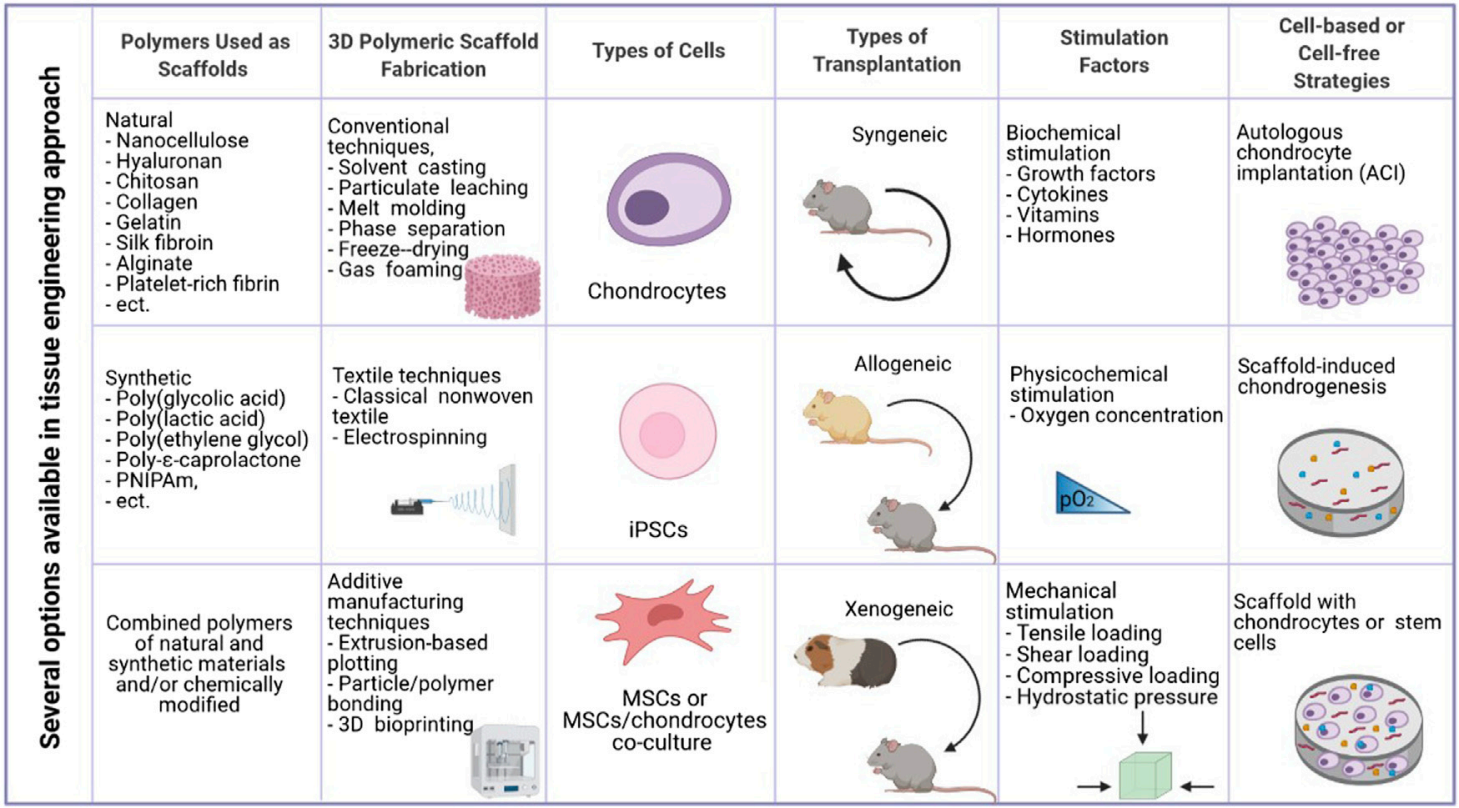
Essential elements in cartilage tissue engineering (created in BioRender.com).

Given the limited spontaneous repair after cartilage injury, tissue engineering approaches for cartilage regeneration are becoming more common (Figure 1). Current cartilage TE techniques include two approaches. The first one involves the transplantation of natural tissue similar to cartilage. Autologous cell implantation is the most popular where patients’ cells are collected from other body parts and located in lesions (Peterson et al., 2010). An obvious limitation of this method is connected with lowering cell viability with aging (Loeser et al., 2016). The second approach involves the fabrication of artificial cartilage with biochemical, biomechanical, and structural properties resembling natural tissue (Walter et al., 2019). Moreover, there exist two different methodologies for fabrication the scaffolding component. The matrix-induced chondrogenesis uses a cell-free material that is placed in the cavity. Additionally, this material could be loaded with various growth factors or cytokines, enhancing repairing and differentiation of chondrocytes (Benthien and Behrens, 2011). Implantation of scaffolds with cells is more challenging but provides more promising results (Makris et al., 2015). To regenerate cartilage, a vast amount of biomaterials in the form of sponges, hydrogels, electrospun fibers, and microparticles have been produced, each of which has unique properties for the stimulation of chondrogenesis (Ahmed and Hincke, 2010; Vega et al., 2017). Although some of the implants produced so far show promising properties and the mere production of artificial cartilage was initially considered an easy goal by scientists, few of them were used to promote cartilage tissue regeneration in the clinic (Ge et al., 2012).

## Advantages and Disadvantages of Nanocellulose Scaffolds

Nanocellulose is the most abundant and renewable material on the planet. Its abundance and renewability, relatively low-cost, and relative ease of availability, among other characteristics, makes NC an excellent biomaterial for constructing scaffolds in cartilage tissue engineering. NC used as a biomaterial is divided into three categories, namely cellulose nanocrystals (CNCs), nanofibrillated cellulose (NFC), and bacterial nanocellulose (BNC). As a polymer composed of glucose subunits, nanocellulose has a wide range of tunable physical, chemical, and biological properties. Hydrophilicity, biocompatibility, and low immunogenicity are the most prominent attributes essential in cartilage TE (Chinta et al., 2021). In general, all types of NC are considered biocompatible since they are nontoxic, nonimmunogenic, noninflammatory, and facilitate cells to adhere, proliferate, migrate, and differentiate, either alone or in composites with other materials (Eslahi et al., 2019). However, toxicology studies on nanocellulose-based materials are still in the early stages. Many studies have shown that CNC can cause an inflammatory response, especially after chronic exposure *via* inhalation, with particle morphology and dimensions of CNCs being critical factors affecting the type of innate immune inflammatory responses (Stefaniak et al., 2014; Yanamala et al., 2014). Because of self-aggregation and bioaccumulation, inhaling a lot of NC might cause lung irritation. *In vitro* cytotoxicity studies of CNCs with various cell lines revealed no harmful effects at low doses (∼50 μg/ml), however high concentrations (>∼100 μg/ml) caused cell death and gene expression alterations in mammalian cells.

Cytotoxicity studies with NFCs revealed no indication of toxicity on the cell membrane or DNA proliferation (Jorfi and Foster, 2015). Also, *in vitro* and *in vivo* investigations of BNC revealed no cytotoxicity. BNC did not cause any DNA damage, apoptosis, or necrosis in cells. It seems that BNC is the most biocompatible among all NC types. By introducing alternative chemical groups on nanocellulose’s surface, the pro-inflammatory reaction can be considerably reduced or switched into an anti-inflammatory effect. What is more, NC was shown to promote tolerogenic dendritic cells with the ability to generate several regulatory T cell subsets (Čolić et al., 2020). The long-term subcutaneous implantation of BNC did not demonstrate any signs of immunogenicity, inflammation, or formation of exudates around the implant, confirming that BNC does not induce the foreign body reaction and acts as an inert substance (Helenius et al., 2006). Not only 2D but also 3D BNC did not interfere with wound haemostasis *in vivo* and evoked a modest acute inflammatory reaction, neither a foreign body nor chronic inflammatory response, according to *in vivo* investigations (Osorio et al., 2019). Regarding NC biocomopatibility, there are also numerous contradictory results, which are most likely due to differences in the primary source of NC, its preparation, structure and shape, fiber length, contamination with endotoxin and glucans, applied concentrations, cell culture models, and a variety of other factors (Chinta et al., 2021).

The potential cytotoxicity of NC may be affected by its physicochemical properties, such as functionalization with specific chemical groups, as reviewed by Čolić et al. (Čolić et al., 2020). The possibility of surface modification, which in other nanostructures is not easy, is possible due to the presence of numerous hydroxyl groups, which provide the option of modification and exploiting chemical reaction strategies (Tortorella et al., 2020).

The great potential of nanocellulose in cartilage tissue engineering also lies in its large surface area and a high volume ratio allowing for adsorption of a wide range of atoms, ions, and molecules. Along with nanocellulose’s hydrophilic hydroxyl moieties, the cell adhesion mechanism enables cells to adhere to cellulose. A large number of hydroxyl groups is also responsible for its water uptake capacity. However, because of high intermolecular and intramolecular hydrogen bonding of free hydroxyl groups, water solubility of NC is limited. Functional derivatization of the OH groups significantly improved the water solubility of cellulose derivatives such as methylcellulose, ethylcellulose, or hydroxypropyl cellulose (Medronho et al., 2012).

Nanocellulose also provides a high potential due to its excellent mechanical features. The crystalline regions increase cellulose’s stiffness and strength, while the amorphous portions offer flexibility. Nanofibers are highly stiff (even 220 GPa of the elastic modulus) and possess a high tensile strength of ∼10 GPa. What is more, the mechanical strength of NC can be is further increased by developing its composites with materials such as ceramics, nanoparticles, and polymers. Furthermore, the mechanical strength of NC can be increased by incorporating mechanically strong reinforcement materials such as ceramics, nanoparticles, and polymers into its composites (Khan et al., 2021). In addition, nanocellulose can be designed to fabricate products with desired shape structural complexity (Martínez Ávila et al., 2016).

Despite their many advantages, the small pore size is a significant drawback of NC, preventing the infiltration of mammalian cells deep into its matrix. The pore size can be modulated to meet the desired features for cartilage tissue engineering applications. NC needs to be also modified due to the lack of active sites that are required for cell signaling (Chinta et al., 2021).

Also, non-degradability is a crucial factor. A variety of fabrication techniques have been used to overcome these limitations, including electrospinning, photolithography, salt leaching, polymer blending, solvent casting, or a combination of nanofibers and microfibers, to create scaffolds with increased pore size and interconnectivity, which promote cellular migration/infiltration (Chinta et al., 2021).

## Hyaline Cartilage–Components and Chondrogenesis

Cartilage is a specialized connective tissue present in several areas of the body. Hyaline cartilage, usually existing within joints, is the most widespread and is responsible for transferring and relieving stress between one bone and the other. Due to the high elasticity of the cartilage, the deformations caused by the loads transferred during the movements of the joints are reversible. Cartilage and synovial fluid form a layer of several millimeters, which significantly reduces the friction between the moving elements. Due to its density, cartilage, unlike most tissues, is not penetrated by blood and lymph vessels or nerves. Chondrocytes, therefore, usually function under low oxygen tension, and nutrients reach the cells *via* the synovial fluid (Eschweiler et al., 2021).

The cartilage extracellular matrix consists of water (60–80% of total weight), collagen proteins (60% dry weight), and proteoglycan aggregates (30% dry weight). Other substances such as lipids, phospholipids, non-collagen proteins and other glycoproteins are the remainder (Sophia Fox et al., 2009). Type II collagen is the most abundant isoform in articular cartilage, which accounts for over 80% of all collagens and, together with aggrecan, is considered a marker of differentiated chondrocytes. This isoform confers resistance to compressive forces in cartilage. Thus, its role in maintaining ECM homeostasis is critical, and loss causes perturbations in cartilage’s physical and mechanical properties, leading to osteoarthritis. The remaining isoforms include collagen type IX and XI (3–15%) and other types (III, VI, X, XII, XIV). Type IX and XI collagens are responsible for the organization and structure of fibers (Sophia Fox et al., 2009; Demoor et al., 2014). Proteoglycans are the second largest group of macromolecules in the ECM of the articular cartilage. They consist of a core protein with one or more covalently attached linear glycosaminoglycan (GAG) chains. Aggrecan, a cartilage-specific proteoglycan, interacts with GAGs and hyaluronan to provide the cartilage its compressive resistance and shock-absorbing capacity under loading. Sulfonated GAG (sGAG) chains, such as chondroitin sulfate, keratan, and dermatan sulfate, are responsible for the ability to store a significant amount of water, ensuring flexibility and viscoelasticity (Demoor et al., 2014). As the adult articular cartilage is avascular, the ECM components with growth factors and cytokines play a crucial role in regulating chondrocyte metabolism and phenotype (Peng et al., 2021).

Various chemical factors, such as soluble growth factors or chemokines, have been shown to affect differentiation of mesenchymal stem cells (MSCs) into chondrocytes. In particular, transforming growth factor β (TGF-β), bone morphogenetic protein (BMP), fibroblast growth factor (FGF), and insulin-like growth factor (IGF) have been demonstrated to induce chondrogenesis in many studies (Augustyniak et al., 2015). Also, small nonprotein molecules, such as a heterocyclic-structured kartogenin (KGN), can induce MSC differentiation into chondrocytes by activating JNK/RUNX1 pathways and suppression of β-catenin/RUNX2 signaling pathways (Jing et al., 2019). Furthermore, by modulating the transcription factor core binding factor beta subunit (CBFB)-RUNX1 signaling, KGN binds filamin A triggering cartilage development.

**FIGURE 2:**
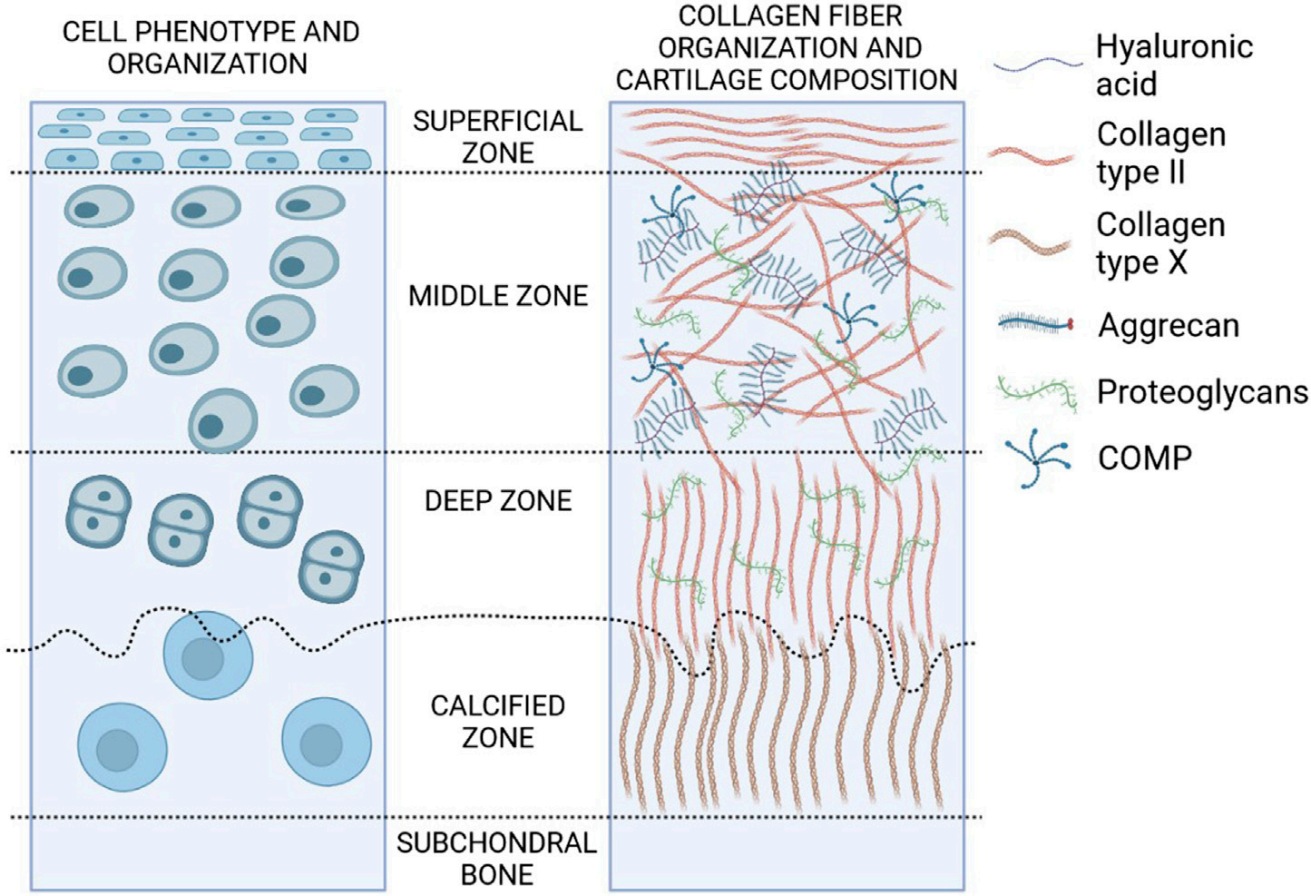
Structure and composition of hyaline cartilage. Zones of hyaline cartilage with varying cellular distribution, shape, collagen organization, and biochemical markers are depicted in this diagram (created in BioRender.com).

Mature hyaline cartilage is structurally and functionally heterogeneous and organized in four distinct areas based on the cell shape, mutual position, and metabolic activity (Figure 2). The first superficial zone (up to 10–20% of the cartilage thickness) contains small fibroblast-like cells and numerous collagen fibers (primarily type II and IX collagen) packed tightly and aligned parallel to the articular surface. This arrangement ensures high tensile strength, necessary in the presence of stress under joint loads. An intermediate zone below (20–70% of the thickness) with chondrocytes, spherical and irregularly distributed, contains proteoglycans and thicker collagen fibrils. The radial zone (up to 70–100% of the thickness) contains chondrocytes arranged in groups perpendicular to the articular surface, the largest diameter collagen fibrils in a radial disposition, the highest proteoglycan content, and the lowest water concentration. Finally, the calcified zone anchors the collagen fibrils of the deep area to subchondral bone. In this zone the cell population is scarce and chondrocytes are hypertrophic (Ge et al., 2012).

**FIGURE 3:**
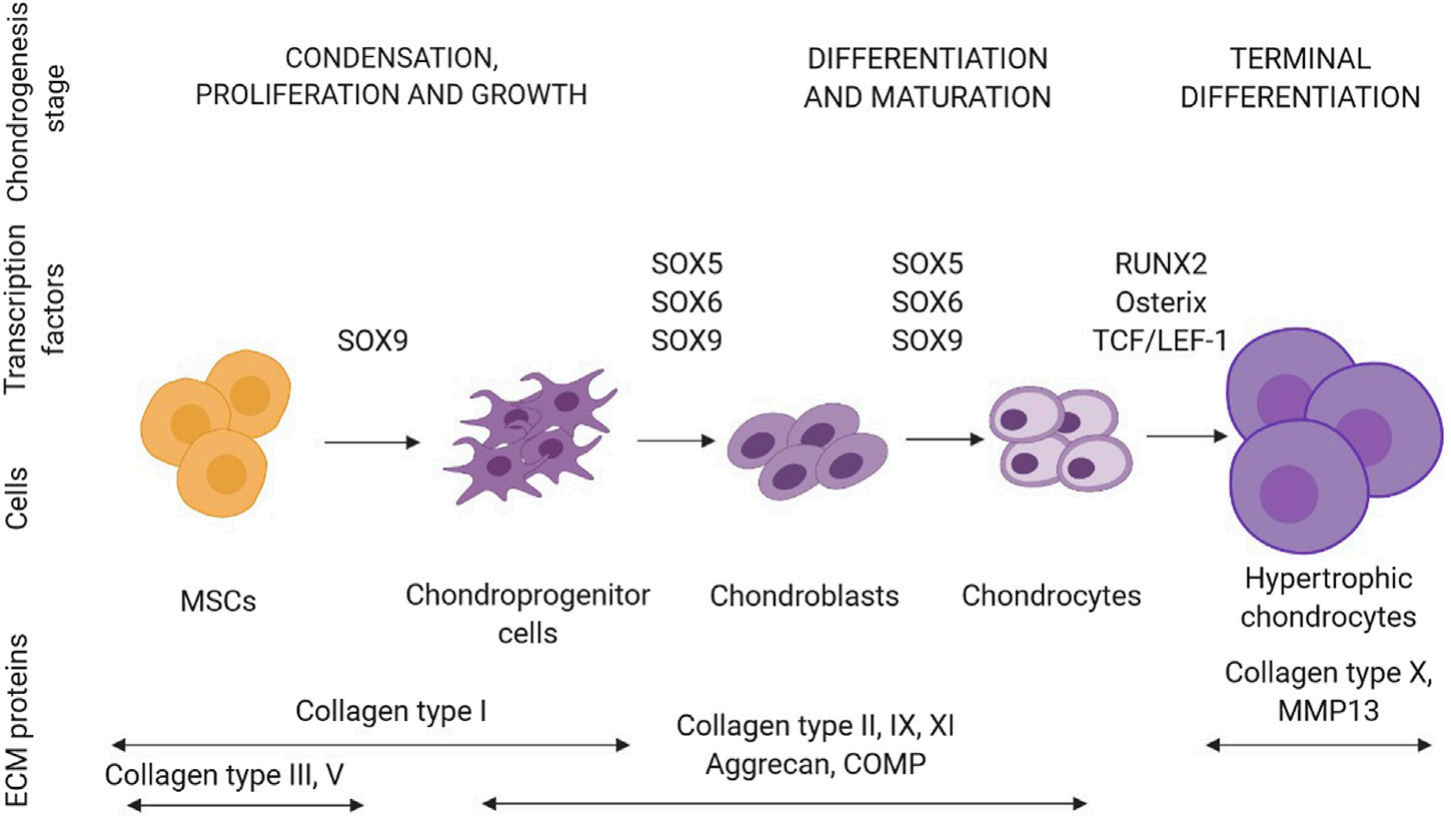
Schematic representation of a sequence of events leading to the differentiation of mesenchymal stem cells (MSCs) towards chondrocytes (created in BioRender.com). In each stage of chondrogenesis, the temporal expression profiles of ECM proteins and transcription factors are highlighted. Abbreviations: COMP, cartilage oligomeric protein.

Cartilage is formed during chondrogenesis in the early phase of embryonic skeletogenesis with condensation of mesenchymal stem cells, which express mainly type I, III, and V collagens, and chondroprogenitor cell differentiation (Figure 3). Interactions between progenitors and ECM lead to differentiation into chondrocytes and synthesis of cartilage-specific ECM components such as collagen type IIB, IX, XI, and aggrecan. The hypertrophic process begins when chondrocytes enlarge and ECM is enriched in type X collagen. SOX9 (SRY-Box Transcription Factor 9) and RUNX2 (Runt-related transcription factor 2) are two transcriptional regulators essential for articular cartilage formation and hypertrophic maturation, respectively. SOX9 binds to chondrocyte-specific enhancer elements of cartilage matrix genes, including *COL2A1*, *COL11A2*, *ACAN* encoding collagen type II α1 chain; collagen type XI α2 chain and aggrecan, respectively. In chondrocytes, *COL10A1,* and *MMP13*, encoding collagen type X α1 chain and matrix metalloprotease 13 - markers of chondrocyte hypertrophy and maturation are direct target genes of RUNX2. Additionally, chondrogenesis is regulated by the interplay of TGF-β, FGF, BMP, and WNT signaling pathways (Goldring, 2012).

## Cell Sources in Cartilage Tissue Engineering

Chondrocytes, the main cellular component of cartilage, form a small percentage (1–10%) of the total volume of cartilage. Therefore, those cells do not exhibit cell-cell contacts. Additionally, mature chondrocytes lack mitotic activity and have a low metabolism that aims to maintain a balance between anabolism and catabolism processes, resulting in ECM components turnover. Collagen type II, aggrecan, and SOX9 are commonly expressed in normal and healthy chondrocytes in their native 3D environment. On the other hand, hypertrophic chondrocytes have a high level of collagen type X, while dedifferentiated chondrocytes synthesize collagen type I. Gelsolin and TGF-β3 secretion are increased in osteoarthritic chondrocytes (Brose et al., 2015).

The majority of cartilage regenerative medicine approaches rely on the implantation of highly differentiated chondrocytes. However, due to limited sources and complex harvesting methods, expansion of isolated cells *in vitro* is needed to obtain sufficient cellular material before re-implantation. This is done in a two-dimensional (2D) setting, where cells dedifferentiate and lose their natural phenotype, resulting in formation of fibroelastic cartilage that contains more collagen type I rather than collagen type II or undergo hypertrophy. Other potential shortcomings of autologous chondrocytes include donor site morbidity and poor capability for intrinsic repair. In addition, changes in the structure of the F-actin cytoskeleton, which also regulates cell mechanical properties, are linked to the loss of chondrocyte phenotype (Darling et al., 2009). Gene (mainly *COL1A1*, *COL2A1*, *COL10A1*, *ACAN*, *SOX9, RUNX2, ALP, VEGF, MMP13*) and protein expression (aggrecan, type I and II collagens), ECM composition (GAG), and cell morphology have been used to determine the differentiation status of isolated chondrocytes. As dedifferentiation progresses, GAGs, *COL2A1*, *SOX9,* and *ACAN* lose their expression (Chen et al., 2015). Notably, the isolated chondrocytes can re-differentiate when inserted into a 3D environment. Chondrocytes are embedded in agarose, alginate beads, polymer gels or compressed into scaffold-free pellets by centrifugation to create 3D cultures. Additionally, using specific medium supplements (*e.g*., ascorbic acid, TGF isoforms, insulin, transferrin, selenite, and others) may support redifferentiation. Consequently, it is essential to develop scaffolds that can be fabricated into porous structures that allow cells to enter the 3D system and stimulate them to return to their differentiated state, where articular cartilage is generated (Caron et al., 2012).

Besides differentiated chondrocytes, cartilage tissue engineering uses chondrogenic progenitors. A pool of undifferentiated progenitor cells with high regenerative potential, limitless division capacity, self-renewal capability, simple accessibility, and hypoimmunogenicity would be ideal source to replace chondrocytes. Various stem cell sources have been studied, including bone marrow, adipose tissue, synovia, umbilical cord blood, periosteum, dental pulp, placenta, and embryos. The use of multipotent mesenchymal stem cells, which have the ability to differentiate into chondrocytes when given the right environmental factors, is an effective and safe way to facilitate chondrogenesis. MSCs should meet the following basic requirements, according to the International Society for Cellular Therapy: adherence to the plastic surface when cultured in standard 2D conditions, expression of CD105, CD73, and CD90 but not CD45, CD34, CD14 or CD11b, CD79, or CD19 and HLA (human leukocyte antigen) class II, the capability of *in vitro* differentiation into osteoblasts, chondroblasts, or adipocytes (Dominici et al., 2006). Bone marrow (BM-MSCs) and stem cells from adipose tissue (ATSCs) are the most commonly used sources of human adult MSCs (Brose et al., 2015). The capacity of MSCs to differentiate into chondrocytes varies, with synovial MSCs showing the most significant potential to differentiate into articular chondrocytes (Zha et al., 2021). For allogenenic mesenchymal stem cell therapy, the umbilical cord may be an appealing source. Wharton’s Jelly, a gelatinous tissue surrounding umbilical vessels, is used to separate umbilical cord MSCs (UC-MSCs). Compared to the gold standard BM-MSCs, they have numerous advantages. Umbilical cords are readily available discarded tissues and the harvesting process is noninvasive. The doubling time of UC-MSCs is at least twice shorter because their properties are located between embryonic stem cells (ESCs) and adult MSCs. Furthermore, long-term *in vitro* culture seems to have little effect on their phenotype or genetic stability and does not induce tumorigenesis. UC-MSCs do not express hematopoietic markers such as CD34 and CD45 and are much more capable for chondrogenic differentiation than BM-MSCs (Mebarki et al., 2021).

Unlike mature chondrocytes, MSCs can be expanded *ex vivo* to yield large numbers of cells, making them a plentiful cell source for autologous cell therapies. Unfortunately, obtaining a pure population of stem cells and precisely controlling the path of MSC differentiation is difficult. Besides, during chondrogenic induction, MSCs appear to develop hypertrophic properties, indicating the possibility of further differentiation toward endochondral bone formation (Arora et al., 2017). To overcome these limitations, cocultures of isolated MSCs and mature chondrocytes were employed to mimic physiological differentiation and regeneration processes. Cocultures tend to be more effective than monocultures, including paracrine interactions between both populations through direct or indirect contact, increased chondrogenic differentiation of stem cell populations, reduced levels of hypertrophy, and facilitated *in vivo* cartilage regeneration (Cho et al., 2018). Furthermore, when chondrocytes were cocultured with MSCs, they were able to maintain a healthy mature phenotype with lower expression of hypertrophic and fibrotic markers (Maumus et al., 2013).

Many of the problems listed above could be solved using induced pluripotent stem cells (iPSCs). In terms of pluripotency, phenotype, and proliferation potential, iPSCs resemble ESCs. However, iPSCs are isolated from a patient’s somatic cells, avoiding the ethical concerns associated with the use of ESCs. The expression of genes related to stemness, such as the combination of *OCT4, SOX2*, *KLF4*, *C-MYC* and *OCT4*, *SOX2*, *NANOG*, and *LIN28*, is needed for the development of iPSCs (Wuputra et al., 2020). Several studies have successfully demonstrated the ability of iPSCs to differentiate into chondrocytes in the presence of the culture media containing growth factors such as TGF-β, FGF, BMP, and WNT. Now, major chondrogenic differentiation approaches of iPSCs include developing MSC-like iPSCs following differentiation into chondrocytes, the process of embryoid body formation, and cocultures of iPSCs-derived MSCs with primary chondrocytes (Csobonyeiova et al., 2021). However, until now, complete reprogramming and differentiation process of iPSCs into chondrocytes still take a long time. Besides, transplanting incorrectly reprogrammed iPSCs carries the risk of tumor formation (Wuputra et al., 2020).

## Sources of Nanocellulose for Cartilage Engineering

Many natural materials have been used to provide a satisfactory bioactive environment and mechanical support to promote the growth of new chondral tissue due to their superior biocompatibility, biodegradability, minimal negative immunological effect, and favorable cellular interaction. When natural polymers are inserted *in vivo*, they exhibit structural compatibility to biological molecules found in animals, minimizing the probability of an immune response. As a result, opposed to synthetic polymers, these materials are either non-immunogenic or have low immunogenicity (Chinta et al., 2021). Examples of natural polymers used in TE for cartilage regeneration include chitosan, collagen, alginate, silk fibroin, hyaluronan, gelatin, and cellulose (Figure 1).

**FIGURE 4:**
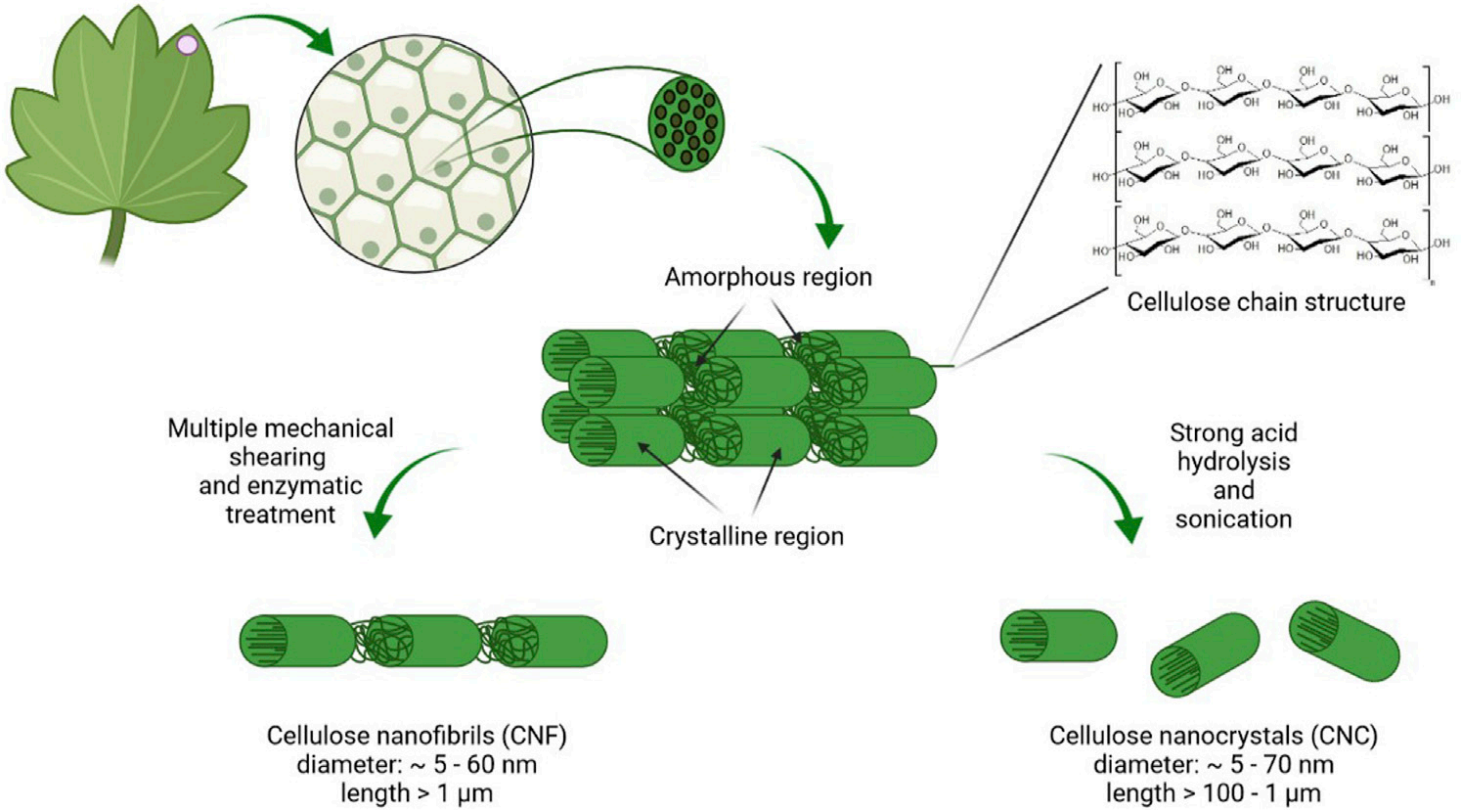
Scheme of plant nanocellulose origin, composition, and organization. Cellulose in plant cells wall is organized into micro- and macrofibrilles. During the purification process lignin and hemicellulose are removed. Acid hydrolysis leads to formation of highly crystalline CNC while NFC is prepared by disintegration with high shear forces (created in BioRender.com).

Cellulose, a linear polysaccharide composed of d-glucopyranose units connected by β-1,4 glycosidic bonds, is the most abundant, sustainable, and renewable material. Cellulose is synthesized by plants, algae, some animals (*e.g*., tunicates), fungi, and bacteria. Among the bacteria, *Komagataeibacter*, *Pseudomonas*, *Agrobacterium*, and *Sarcina* can synthesize BNC from glucose and other carbon sources. The reinforcement segments in cellulosic fibrils in plants, algae, and bacteria are made up of highly organized (crystalline) and disordered (amorphous) microfibrillar motifs in an alternating arrangement (Figure 4). This typical configuration gives cellulose its essential mechanical behavior, with the amorphous motifs providing flexibility and plasticity and the crystalline motifs providing the strength and elasticity. The geometry of the arrays of cellulose synthases that assemble them determines the thickness and structure of cellulose microfibrils (Dutta et al., 2019).

The biosynthesis mechanisms of a plant (CNC and NFC) and bacterial (BNC) NC varies significantly. The top-down approach, in which chemical/mechanical breakdown methods disrupt the lignocellulosic biomass, is commonly used to synthesize plant cellulose from wood, cotton, hemp, potato tubers, and algae. Plant NC is manufactured from natural cellulosic materials using processes that preserve much of the cellulose microfibrils’ native structure. Native cellulose fibers are commonly found in the plant cell wall as a quasi-solid, cohesive framework. The diameter of cellulose microfibrils in higher plants is typically about 3 nm and the microfibrils are aggregated into bundles that are 10–20 nm thick (Jarvis, 2018). NFC, usually produced by delamination of wood pulp by mechanical pressure before and/or after chemical or enzymatic treatment, contains microfibrils with a diameter of 5–60 nm and length of several micrometers. CNC, also known as whiskers, contains rodlike cellulose crystals with widths and lengths ranging from 5 to 70 and 100 nm to several micrometers, respectively. Acid hydrolysis is used to remove amorphous portions of a purified cellulose source, accompanied by ultrasonic treatment (Klemm et al., 2011).

BNC pellicles are produced biotechnologically using a variety of bacteria (including *Komagataeibacter*) as an exopolysaccharide at an air-tight interface under static culture conditions (Mahendiran et al., 2021). The bacteria’s cellulose fibrils, joined to form ribbons and then a 3D nanofiber network (fiber diameter below100 nm). The hydrogels of BNC possess up to 99% water. BNC was found to be very pure with a high Mw (weight-average molecular weight), high crystallinity, and good mechanical stability. The biofabrication method opens up the exciting possibility of producing cellulose through fermentation and controlling the shape and the structure of the nanofiber network during biosynthesis (Klemm et al., 2011).

The physical properties of nanocellulose are determined by three hydroxyl groups at the C-2, C-3, and C-6 positions. Due to the presence of reactive surfaces with OH group various derivatives can be fabricated by introducing functional groups using chemical, mechanical, and/or enzymatic methods. The presence of three hydroxyl groups per repeating unit also makes NC a strong candidate for initiating chondrogenesis because the presence of OH groups on a biomaterial’s surface has been shown to promote stem cell chondrogenic differentiation (Curran et al., 2005). Therefore, there are many examples of NC-based scaffolds inducing stem cells to differentiate into chondrocyte phenotypes and then promoting the development of cartilage tissue (*e.g*., Taokaew et al., 2014; Chen et al., 2018; Talaat et al., 2020).

Nanocellulose used in cartilage TE is available in different formats like hydrogels, membrane-like structures, porous membranes, electrospun fibers, porous scaffolds, and bioinks (Tables 1 – 3). Hydrogels are the most widely used biopolymers in cartilage engineering because of their highly swollen 3D environment, which is very similar to soft tissues and allows for the diffusion of nutrients, growth factors, and cellular waste through the elastic network. A hydrogel is a soft material made up of a porous 3D network of crosslinked polymer chains that can hold up to 99.9% water. 3D hydrogels, unlike standard 2D culture, allow chondrocytes to preserve their shape and phenotype while providing a physiological environment in which they can form cartilage tissue. The gelatinous and flexible 3D scaffold is necessary to sustain cell growth and mimic the niche observed *in vivo*. Hydrogels were first used in orthopedic applications for the less invasive treatment of localized cartilage defects *via* injection or arthroscopy (Liu et al., 2017). Hydrogels that mimic the properties of ECM have shown high potential as tissue engineering scaffolds and injectable hydrogels. Mixing therapeutic and bioactive signaling agents and cells with precursor solutions is a simple way to introduce them into scaffolds (Aswathy et al., 2020). Nanocellulose-based hydrogels can be made from bacterial and plant NC, such as CNC, NFC, and BNC, and cellulose derivatives such as methylcellulose (MC), carboxymethylcellulose (CMC), and hydroxypropylmethylcellulose (HPMC). Physical cross-linking in the presence of triggers (temperature, ions, pH) or chemical cross-linking (free radical polymerization, Michael-type addition, Schiff’s base reaction, enzymatic cross-linking, *etc*.) are used to create a cellulose-based hydrogel by *in situ* gelation (Balakrishnan et al., 2013).

**TABLE 1:** Plant-derived NC applied to cartilage tissue engineering.

Plant NC type	System	Source of chondrocytes or stem cells and experimental design	Outcome	Analyses related to differentiation and proliferation	Reference
Hydrogels					
Cellulose hydrogel	Filter paper pulp dissolved in heated LiBr; microporous scaffolds prepared with dispersed NaCl particles	Rabbit chondrocytes	*In vitro*	sGAGs	Isobe et al. (2018)
			Chondrocytes formed 3D spheroid structures on the surface of sponge-like cellulose gel matrix		
CNC/USPIO/SF hydrogels	Hydrogels prepared by mixing CNC/USPIO with SF at room temperature and lyophilization	BM-MSCs were seeded on the hydrogels for 4 and 14 days	*In vitro*	Cell viability, gene expression (*COL1A1*, *SOX9*, *COL2A1*, *ACAN*), ferric irons, proteoglycans, multiparametric MRI	Chen et al. (2018)
			Proliferation of BM-MSCs was not affected by USPIO labeling. A spheroid shape with ECM deposition was observed. CNC/USPIO/SF did not promote the fibrocartilage phenotype		
		The hydrogels were implanted in the cartilage defects of adult rabbits for 4, 8, and 12 weeks	*In vitro*		
			There was no difference in biocompatibility, chondrogenesis, and regeneration efficiency in the groups implanted with non-labeled or USPIO-labeled BM-MSC-loaded hydrogels		
CNC/Dex/USPIO/KGN hydrogels	CNC/Dex/USPIO/KGN hydrogels prepared by injecting the USPIO-KGN solution into CNC/Dex hydrogels followed by drying at room temperature	BM-MSCs were seeded on the hydrogels for 14 days The hydrogels were implanted in the cartilage defects created on the center of the trochlear groove of adult rabbits for 6 and 12 weeks	*In vitro*	Cell proliferation and viability, SEM, H&E staining, proteoglycans, sGAGs, collagen I, II, gene expression (*SOX9*, *COL1A1*, *COL2A1*, *ACAN*)	Yang et al. (2019a)
			Higher proliferation, differentiation and secreted ECM on CNC/Dex/USPIO/KGN as compared to hydrogels without KGN		
			*In vivo*		
			The CNC/Dex/USPIO-KGN group had the best cartilage regeneration and the defect was mostly repaired after the 6th week		
CNC/collagen/USPIO/KGN scaffolds	Scaffolds prepared by injecting the USPIO-KGN solution into the collagen/CNC scaffolds followed by lyophilization	BM-MSCs were seeded on the cylinder scaffold samples and cultured up to 14 days	*In vitro*	Cytotoxicity, cell viability, SEM, proteoglycans, DNA content, gene expression (*SOX9*, *COL1A1*, *COL2A1*, *ACAN*)	Yang et al. (2019b)
			Scaffolds were cytocompatible. High expression of *SOX9* and *COL1A1* could cause a tendency to induce formation of fibrotic cartilage instead of hyaline cartilage. Furthermore, a CNC-enhanced collagen scaffold with higher mechanical properties could cause mechanical stress damage *in vivo*		
a-CNCs/collagen hydrogel	a-CNCs produced through oxidation. aCNC/collagen hydrogel crosslinked by Schiff base bonds	BM-MSCs from neonatal rabbits were mixed with a-CNC/collagen suspension to prepare BM-MSC-loaded discs Hydrogels with BM-MSCs were injected subcutaneously to the mid-dorsal region of nude mice for 1 and 3 days	*In vitro*	Cell viability, confocal microscope, H&E staining	Zhang et al. (2020)
			BM-MSCs encapsulated in the a-CNC/collagen hydrogel showed high viability		
			*In vivo*		
			Subcutaneous injection showed improved implant integrity and higher cell retention		
PLA/S-CNC/P-CNC multilayer scaffold	A porous multilayer scaffold prepared by wetting the surface of one of the layers with a dust-free tissue paper impregnated with acetone and bring it into contact with a second layer	Human fetal chondrocytes were seeded on cylindrical scaffolds (8 mm diameter and ∽ 5 mm thickness) for 2 or 4 weeks or on non-porous films PLA/S-CNC and PLA/P-CNC nanocomposites films and cultured for 7 days	*In vitro*	Cell viability, cytoskeleton imaging, LDH assay, the protein content, proteoglycans, gene expression (*COL1A1*, *COL2A1*)	Camarero-Espinosa et al. (2016)
			The biocompatibility of the three individual layers by culturing chondrocytes on non-porous films was confirmed		
			Chondrocytes grown in the oriented PLA superficial layer were elongated, in isotropic PLA/S-CNC middle layer - rounded, and in the oriented PLA/P-CNC deep layer- elliptical		
CNC/alginate, NFC/alginate, NCB/alginate hydrogels	NFC, CNC and a blend (NCB) produced as an aqueous slurry from raw wood biomass with AVAP^®^ technology and mixed with alginate	hNCs were mixed with the hydrogels to prepare pellets and cultured for up to 7 days	*In vitro*	Cell viability	Al-Sabah et al. (2019)
			Cell viability was minimally affected in autoclave and UV-sterilized, apart from the cells present in ethanol-sterilized hydrogels; the NC-based hydrogels had average pore sizes ranging from 0.22 to 0.91 μm, depending on the NC form assessed		
PCL/PAA/CNC hydrogel	PCL/PAA/CNC fabricated through condensation and addition polymerization	Fibroblast cells (SNL76/7) seeded on PCL/PAA/CNC hydrogels for 3 days	*In vitro*	Cell viability	Pourbashir et al. (2020)
			The incorporation of an optimized amount of CNC (0.5%) improved the mechanical properties of artificial cartilage. Viability testing and cell attachment confirmed the biocompatibility of the material		
NFC/CS/GP hydrogel	NFC from hemp hurd added to GP/CS to form the hydrogel-forming solutions	hDPSCs and BM-MSCs mixed with NFC/CS/GP and cultured for 21 days Chondrogenesis of hDPSCs was compared to BM-MSCs The hydrogel with embedded hDPSCs was injected subcutaneously into rats up to 21 days	*In vitro*	Cell viability, H&E, collagen II staining, gene expression (*COL2A1*, *COL10A1*, *ACAN*)	Talaat et al. (2020)
			CS/GP with CS to GP w/w ratio of 2:25 with good stability in the culture media was chosen. Viability of hDPSCs was slightly lower than BM-MSCs		
			*In vivo*		
			The chondrogenic potential of hDPSCs in NFC/CS/GP was shown to be similar to BM-MSCs		
NFC/alginate bioink	CELLINK^®^	hNCs were encapsulated in bioink formulation [80:20 NFC/alginate (w/w)] and 3D bioprinted cell-laden gridded constructs were analyzed after day 1 and 7 of culture	*In vitro*	Indirect cytotoxicity test, viability, proteoglycans, mineralization, collagen I, II, X; DNA, sGAGs, collagen contents	Markstedt et al. (2015)
			Higher viability was observed before embedding compared to the printed constructs after 1 day (the preparation and mixing caused a decrease in viability). An increase in cell viability was detected in the printed constructs after 7 days of 3D culture compared to day 1		
NFC/alginate bioink	CELLINK^®^	hNCs and rabbit auricular chondrocytes (rACs) were mixed with NFC/alginate and cell-laden bioink was printed into auricular and lattice structures and cultured for up to 28 days	*In vitro*	Cytotoxicity, viability, cytoskeleton imaging, electron microscopy, DNA, GAG content, gelatine zymography, gene expression (*COL1A1*, *COL2A1*, *VCAN*, *RUNX2*, *SOX9*, *COMP*, *MATN3*, *RUNX2, ACAN* ), H&E, COMP, collagen II, GAG, aggrecan, matrilin-3 staining	Martínez Ávila et al. (2016)
			The NFC/alginate increased redifferentiation, proliferation, viability		
NFC/alginate bioink	CELLINK^®^	hBM-MSCs and/or hNCs were mixed with NFC/alginate and cell-laden bioink was bioprinted as 3D gridded constructs. Constructs were implanted in a subcutaneous pocket on the back of the mice up to 60 days	*In vivo*	GAGs, collagens staining, collagen II, FISH	Möller et al. (2017)
			The combination of NFC/alginate, hNCs, and hBM-MSCs for 3D bioprinting resulted in high-fidelity constructs with good mechanical properties and a gradual increase of GAGs. The cocultured group showed a more pronounced cell proliferation and enhanced deposition of human collagen II		
NFC/alginate bioink	CELLINK^®^	hBM-MSCs and/or hNCs were mixed with NFC/alginate and bioprinted into the 3D-grid constructs (5 × 5 × 1.2 mm); the printed constructs were then implanted into a subcutaneous pocket on the back of nude Balb/C mice up to 60 days	*In vivo*	Proteoglycans, collagens, collagen II, Ki-67 staining, FISH	Apelgren et al. (2017)
			Constructs consisting of hNCs showed good proliferative capacity and cartilage-cluster formation. In constructs comprising a mixture of chondrocytes and stem cells, an additional proliferative effect was observed involving GAGs and type II collagen synthesis. The density of GAG-positive cells in the mixed constructs was lower than in constructs with only chondrocytes		
NFC/alginate bioink	CELLINK^®^	hBM-MSCs and hNCs with NFC/alginate were bioprinted into the 3D grid constructs (10 × 10 × 1.2 mm); Constructs were implanted into a subcutaneous pocket on the back of nude mice. After 45 days, a full-thickness skin allograft was transplanted onto the constructs and the construct enclosed subcutaneously. Group 1 was sacrificed on day 60, whereas group 2 had their skin-bearing construct uncovered on day 60 and were sacrificed on day 75	*In vivo*	GAGs, collagens staining	Apelgren et al. (2018)
			The skin transplants integrated well with the 3D bioprinted constructs. A tight connection between the fibrous, vascularized capsule surrounding the 3D bioprinted constructs and the skin graft was observed. The skin grafts survived the uncovering and exposure to the environment		
NFC/alginate bioink	CELLINK^®^	hNCs and hBM-MSCs or hNCs and human SVF (stromal vascular fraction)-derived stem cells from abdominal lipoaspirate mixed with NFC/alginate bioink were 3D bioprinted into 6 mm × 6 mm × 1.2 mm grid and implanted subcutaneously on the back of nude mice up to 10 months	*In vivo*	GAGs, proteoglycans, the number of chondrocytes	Apelgren et al. (2021)
			The transplanted human chondrocytes proliferated in clusters with the formation of cartilage-like tissues. Over the 10-months, no adverse events were observed. SVF-derived stem cells exerted similar trophic effects on chondrocyte proliferation to those observed using hBM-MSCs		
NFC/alginate and NFC/HA bioinks	CELLINK^®^ or NFC was mixed with HA-based hydrogel	Human iPSCs generated from chondrocytes using mRNA-based reprogramming and/or irradiated were 3D bioprinted into cartilage mimics using NFC/alginate and NFC/HA composite bioink and cultured up to 5 weeks	*In vitro*	H&E, GAGs, collagens, proteoglycans, collagen II, Oct4 staining, gene expression (*ACAN*, *SOX9, COL2A1*), cytoskeleton imaging, two-photon excitation fluorescence and second-harmonic generation	Nguyen et al. (2017)
			The NFC/HA bioink showed low proliferation of the limited cell population remaining in the construct after encapsulation and phenotypic changes away from pluripotency. NFC/alginate (60/40, dry weight % ratio) maintained a pluripotent phenotype after 3D bioprinting. After 5 weeks, hyaline-like cartilaginous tissue with collagen type II expression and lacking tumorigenic Oct4 expression was observed in NFC/alginate		
NFC/alginate sulfate bioink	NFC was mixed with alginate sulfate	Chondrocytes isolated from articular cartilage of calf condyle were encapsulated into NFC/alginate sulfate gel discs and cultured up to 28 days or bioprinted into grid-like constructs	*In vitro*	Cell viability, cytoskeleton imaging, collagen I, II, proteoglycan hyaluronic acid-binding region staining	Müller et al. (2017)
			Chondrocytes in NFC/alginate sulfate gel discs were viable, mitogenic and synthesized collagen II. The printing conditions affected the behavior of the cells; however, with wide diameter, conical needles providing the best preservation of cell function		
CNC/alginate, NFC/alginate, NCB/alginate bioinks	NFC, CNC and a blend (NCB) produced as an aqueous slurry from raw wood biomass with AVAP^®^ technology and mixed with alginate	Chondrocytes isolated from human nasoseptal cartilage samples were mixed with NC/alginate bioinks, 3D bioprinted into cylinder discs (5 mm diameter and 4 mm height) and cultured up to 3 weeks	*In vitro*	Cell viability, LDH release, SEM, compression testing	Jessop et al. (2019)
			NC was non-cytotoxic with no significant differences in cell viability post-printing with CNC, NFC and NCB. NCB/alginate formulation was chosen for biocompatibility experiments, where increased viable chondrocytes and maintained a rounded chondrogenic phenotype after prolonged culture		
CNC-GelMA/HAMA hydrogel ink	CNCs were mixed with GelMA and HAMA and LAP as a photoinitiator to form the reinforced hydrogel ink	ATDC5 cells were encapsulated in the optimal GelMA/HAMA ink containing 0.030% (w/v) LAP and the hybrid printed constructs were fabricated. Cell viability was evaluated over 7 days	*In vitro*	Cell viability	Fan et al. (2020)
			The coaxial printing strategy allowed the generation of 3D structures where cells were encapsulated in the core and protected by the shell comprising the crosslinked form of GelMA/HAMA ink. Cells remained viable		
Electrospinning					
Cellulose/SF composite fibers	Cellulose from wood pulp and silk polymer blend (75:25 ratio) was electrospun into nanofibers without post-spun treatment, using a trifluoroacetic acid and acetic acid cosolvent system	hBM-MSCs were seeded on composite nanofibrous discs [with and without fibronectin (FN) coating] and cultured up to 2 weeks	*In vitro*	Cell viability, adhesion, gene expression (*COL1A1*, *COL2A1*, *ACAN*, *SOX9*, *ALPL*, *PPARG*), cytoskeleton imaging paxillin, aggrecan, collagen II staining, chondrogenic signaling	Begum et al. (2020)
			hBM-MSCs maintained similar viability and cell binding with and without FN coating. All chondrogenic genes were upregulated without addition of TGF-β. Cells cultured on the composite substrate showed a lesser cell stretch with diffuse paxillin staining, demonstrating the hMSC response to the elasticity of this surface. The chondroinduction did not involve the main chondrogenic differentiation signaling pathway (Smad2/3 regulated phosphorylation via TGF-β)		
Membranes/other scaffolds					
Paper cellulose membrane	Cellulose membrane chamber from a silicone tube (21 mm × 15 mm × 10 mm, outer diameter × inner diameter × height) and cellulose nanopore-size membrane (*in vivo* bioreactor (IVB)	Human fetal cartilage progenitor cells (hFCPCs) were placed into the IVB and implanted under back skins of rabbits 1 week cultured engineered cartilage tissue from IVB was then implanted into a cartilage defect created in the trochlear groove of the femur of adult rabbits up to 12 weeks	*In vivo*	Cell viability, H&E staining, proteoglycans, DNA, sGAG, collagen contents, mineralization, collagen I, II, X	Li et al. (2020)
			The IVB promoted the synthesis of cartilage matrix and maintained the cartilage phenotype and delayed calcification. The protein, glucose, and HA contents in the IVB bioreactor fluid were closer to synovial fluid than serum		
			The IVB promoted better healing of the cartilage defect		
CaP-coated cellulose	Regenerated cellulose II monofilament fibres were activated in a saturated Ca(OH)_2_ solution and coated with (CaP) carbonated apatite	Chondrocytes isolated from the articular cartilage of bovine calves cultured up to 6 weeks	*In vitro*	Cell viability, proteoglycans, collagen I, II	Müller et al. (2006)
			CaP-coated cellulose was the suitable material in terms of chondrocyte adhesion and cartilage development (vitality of the adhered chondrocytes was excellent but collagen I and II expression was comparable on untreated as well as o CaP-coated scaffolds)		
NFC/PEGDA aerogels	A mixture of NFC and PEGDA used to prepare aerogels with a multiscale pore structure through a combination of stereolithography and freeze-drying	Mouse BM-MSCs were seeded on aerogel scaffolds and cultured for 21 days	*In vitro*	Cell viability, SEM, H&E, proteoglycans, GAGs	Tang et al. (2021)
			Honeycomb structure of the scaffold affected the proliferation of mBMSCs and secretion of proteoglycans		
Cellulose/silk membranes	Blends of wood pulp cellulose and silk were prepared using a ionic liquid (1-ethyl-3-methylimidazolium acetate) as a solvent	hBM-MSCs were seeded on fibronectin-coated materials cellulose/silk circular discs and cultured up to 2 weeks	*In vitro* hBM-MSCs growing on a blend combination of cellulose and silk in a 75:25 ratio upregulated the expression of chondrogenic marker genes in the absence of TGF-β. No adipogenic or osteogenic differentiation was detected	Cell adhesion and viability, gene expression (*ACAN*, *SOX9, COL1A1*, *COL2A1*), triglicerides, collagen II staining	Singh et al. (2013)
NFC/starch composites	NFC extracted from the rice straw with chemo-mechanical method was combined with starch by a combination of film casting, salt leaching, and freeze-drying methods	Chondrocytes isolated from articular cartilage were cultured on NFC/starch scaffolds up to 7 days	*In vitro*	Cell viability, SEM	Nasri-Nasrabadi et al. (2014)
			NFC/starch porous scaffolds with interconnected pores supported attachment and growth of chondrocytes		

**TABLE 2:** Cellulose derivatives applied to cartilage tissue engineering.

Cellulose derivative type	System	Source of chondrocytes or stem cells and experimental design	Outcome	Analyses related to differentiation and proliferation	Reference
Hydrogels					
CMC/sCMC/gelatin hydrogels	CMC, sCMC, and gelatin conjugated with tyramine groups with N-(3-dimethylaminopropyl)-N′-ethylcarbodiimide/N-hydroxysuccinimide	A combination of caprine ACs and IFP-MSCs were encapsulated in the hydrogels and cultured for up to 28 days	*In vitro*	Cell viability, membrane permeabilization, cytoskeleton imaging, proteoglycans, GAGs, DNA and collagen content, collagen I, II, X staining	Arora et al. (2017)
			The encapsulated TGF-β1 was shown to be bioactive and supported better cell survival over control hydrogels. The TGF loaded hydrogels demonstrated good sulfated GAG and expression and deposition of hyaline cartilage markers and reduced the deposition of fibrocartilage and hypertrophy markers		
Oxidized CMC/gelatin hydrogel	Borate–diol complexation of oxidized CMC (dialdehyde derivative CDA) with borax followed by Schiff’s reaction of CDA’s aldehyde groups with the amino groups of gelatin led to the formation of a rapidly *in situ* gelling hydrogel	Murine articular chondrocytes were seeded on the hydrogel and culture for 24 h or encapsulated and incubated 4 weeks A defect was made in a goat patella and filled with hydrogel	*In vitro*	Cytoskeleton imaging, GAGs, DNA content, proteoglycans, collagen II staining, ROS generation	Balakrishnan et al. (2013)
			Hydrogels revealed porous structure with ROS generation		
			*In vivo*		
			The hydrogel integrated well with host cartilage tissue. Chondrocytes existed in clusters, migrated through the scaffold and maintained GAG deposition		
CMCA hydrogel	CMCA obtained by converting a large percentage of carboxylic groups (∼50%) of CMC into amidic groups	Chondrocytes from the human knee articular cartilage were mixed with the hydrogel and cultured for up to 14 days The hydrogels were implanted in the cartilage defects of adult rabbits. The treatments were repeated every 10 days for 30 or 50 days	*In vitro*	Cell viability, collagen II, C-propeptide, aggrecan, IL-1β, MMP-1, MMP-13, proteoglycans, cathepsin B	Leone et al. (2008)
			CMCA supported growth, differentiation, and the synthesis of ECM components. CMCA hydrogel did not stimulate the release of MMP1 and MMP13		
			*In vivo*		
			CMCA stimulated cartilage healing		
PU/MC hydrogel composites	A thermo-reversible hydrogel composed of MC in a Na_2_SO_4_ solution obtained by dispersion technique	hBM-MSCs in a hydrogel were filled into cylindrical (8 mm diameter × 4 mm height) porous PU scaffolds (pore size of 90–300 μm), transferred into a pin-on-ball bioreactor system and subjected to 21 loading cycles 1 cm diameter specimens of the hydrogel were subcutaneously implanted into the dorsal skin of mice for 3 and 6 weeks	*In vitro*	Gene expression (*COL1A1*, *COL2A1*, *ACAN*, *SOX9, COL10A1*), ALP activity, sGAGs, proteoglycans, collagen I, II, X	Cochis et al. (2017)
			Mechanical stimulation led to a significant increase in chondrogenic gene expression, while histological analysis detected sGAGs and collagen II only in loaded specimens		
			*In vivo*		
			The lack of any hydrogel-derived immunoreaction was demonstrated		
Si-HPMC hydrogel	Si-HPMC was synthesized by grafting 3-glycidoxypropyltrimethoxysilane on HPMC in heterogeneous medium	Rabbit articular chondrocytes (shoulders, knee, and femoral heads) and human chondrocyte SW1353 and C28/I2 cell lines 3D cultured in Si-HPMC hydrogel	*In vitro*	Cell viability, proliferation, gene expression (*COL1A*, *COL1A2*, *ACAN*), sGAGs	Vinatier et al. (2005)
			Si-HPMC had no adverse effects on chondrocyte viability and proliferation. Chondrocytes retained viability, developed nodular structures, produced GAGs and expressed chondrocyte markers		
Si-HPMC hydrogel		Human NCs were allowed to attach in wells of culture plates. After 24 h, Si-HPMC was added to each well for 1, 2, and 3 days. Chondrocytes were also mixed with Si-HPMC for 3D culture for 3 weeks hNCs in Si-HPMC hydrogel were implanted subcutaneously in the back along each side of nude mice for 3 weeks	*In vitro*	Cell viability, proliferation, gene expression (*COL1A*, *COL1A2*, *ACAN*), sGAGs, proteoglycans, H&E staining, collagen fibers, Ki67, collagen II	Vinatier et al. (2007)
			Si-HPMC had no adverse effects on hNC viability and proliferation. hNCs produced GAGs, developed nodular structures and expressed chondrocyte markers		
			*In vivo*		
			After implantation, a cartilaginous tissue exhibiting a nodular structure containing GAGs and type II collagen was formed. Chondrocytes were positively stained with Ki-67 antibodies		
Si-HPMC hydrogel		Dedifferentiated rabbit NCs were mixed with Si-HPMC for 3D culture for 3 weeks Si-HPMC hydrogels with NCs were implanted in the cartilage defects created on the center of the femoral trochlea of adult rabbits for 6 weeks	*In vitro*	Gene expression (*COL1A*, *COL1A2*, *ACAN*), sGAGs, H&E staining, proteoglycans, collagen II	Vinatier et al. (2009)
			NCs in 3D exhibited a higher expression of type II collagen and aggrecan and lower expression of collagen I than the 2D cultures		
			*In vivo*		
			A tissue exhibiting the production of GAG, type II collagen and displaying a specific organization resembling native articular cartilage was formed		
Si-HPMC hydrogel		Human ATSCs or freshly isolated horse NCs were mixed with Si-HPMC and implanted in subcutaneous pockets of nude mice for 5 weeks	*In vivo*	GAGs, type II collagen	Merceron et al. (2010)
			The capability of ATSCs to give rise to cartilage when implanted with Si-HPMC hydrogel was shown. Horse NCs served as a positive control		
Si-HPMC hydrogel		Human ATSCs or horse NCs were mixed with Si-HPMC and cultured in the presence of chondrogenic or control medium for 28 days or implanted in subcutaneous pockets of nude mice for 5 weeks	*In vitro*	GAGs, type II collagen, gene expression (*COL1A*, *COL1A2*, *ACAN, COMP*)	Merceron et al. (2011)
			Si-HPMC supported chondrogenic differentiation of ATSCs. 3D pellet culture, but not 2D conditions, in chondrogenic medium supported GAGs and collagen II production		
			*In vivo* hATSCs formed nodules with cartilaginous features		
Si-HPMC with GY785	GY785 was mixed with Si-HPMC to prepare composite hydrogels	Rabbit ACs attached to wells of culture plates and HPMC/GY785 were added for 1, 2, and 3 days Freshly isolated or dedifferentiated ACs were mixed with Si-HPMC/GY785 for 3D culture for 3 weeks Constructs of ACs with Si-HPMC/GY785 were implanted into subcutaneous pockets of nude mice for 3 weeks	*In vitro*	Cell viability, proliferation, GAGs, collagen I, II, aggrecan staining, gene expression (*COL1A*, *COL1A2*, *ACAN*)	Rederstorff et al. (2017)
			GY785 did not affect ACs viability and proliferation		
			Si-HPMC/GY785 hydrogels supported a differentiated chondrocyte-like phenotype		
			*In vivo*		
			Si-HPMC/GY785 constructs transplanted into nude mice revealed the production of a cartilage-like ECM containing high amounts of GAGs and collagen II		
Si-HPMC hydrogel with laponites	Laponites mixed with Si-HPMC to prepare composite hydrogels	Subcutaneous implantation of hNCs in conjunction with Si-HPMC/1% laponites in the back along each side of nude mice for 6 weeks	*In vivo*	H&E staining, sGAGs, proteoglycans	Boyer et al. (2018)
			The composite hydrogel and entrapped hNCs formed a cartilage-like tissue with ECM containing GAG and collagens		
Si-HPMC with Si-chitosan hydrogels	Hydrogels obtained by mixing of Si-HPMC with Si-chitosan	hASCs or canine ACSs attached to the surface of culture plates. After 24 h hydrogel was added up to 7 days. Chondrocytes were also mixed with hydrogel for 3D culture for 7 days. Hydrogel with hASC was implanted into subcutaneous pockets of nude mice for 6 weeks and with canine ASC in the osteochondral defects of dogs for 4 months	*In vitro*	Cell viability, GAGs, H&E staining, proteoglycan, collagen, elastin, muscle, mucin and fibrin, collagen I, II staining	Boyer et al. (2020)
			The addition of Si-chitosan to Si-HPMC did not significantly alter the viability of 3D cultured hASCs		
			*In vivo*		
			In the canine osteochondral defect model, while the empty defects were only partially filled with a fibrous tissue, defects filled with hydrogel with or without ASCs, revealed a significant osteochondral regeneration		
poly (NIPAAm)-*g*-MC copolymer hydrogel	*N*-isopropylacrylamide (NIPAAm) grafted to MC with ammonium persulfate as initiator and N,N,N′,N′-tetramethylethylenediamine as an accelerator	ATDC5 cells were encapsulated at high cell density within the hydrogel and cultured for up to 28 days	*In vitro*	Cell viability, proliferation, H&E, proteoglycan, GAGs	Sá-Lima et al. (2011)
			PNIPAAm-*g*-MC did not affect the cell viability and proliferation and augmented the synthesis of GAGs with round-shaped morphology		
HEC/CS/GP hydrogel	Chitosan-GP was mixed with HEC	A chondral defect was created in the trochlear groove or medial femoral sheep condyle and calf chondrocytes mixed with HEC/CS/GP stained with calcein AM were injected into the spaces between the osteochondral grafts. Articular chondrocyte nuclei were labeled by immersing the entire mosaic arthroplasty with Hoechst 33,342 In live sheep, a full-thickness unilateral defect was created in the medial femoral condyle	*Ex vivo* and *in vivo*	Fluorescent microscopy	Hoemann et al. (2007)
			HEC/CS/GP loaded with viable chondrocytes formed an adhesive seal with *ex vivo* mosaic arthroplasty defects from sheep knee joints. In mosaic arthroplasty defects of live sheep, bleeding occurred beneath part of the hydrogel carrier, and the gel was cleared after 1 month *in vivo*		
HEC/CS/GP hydrogel	Chitosan-GP was mixed with HEC	Chondrocytes isolated from sheep articular cartilage of knee joint were mixed with HEC/CS/GP hydrogel and cultured for 3 weeks or transplanted to the defects created in the knee for 12 and 24 weeks	*In vitro*	Cell viability, H&E, proteoglycan, GAGs, collagen II staining	Hao et al. (2010)
			Chondrocytes in the hydrogels retained normal growth and accumulated pericellular sGAGs and type II collagen		
			*In vivo*		
			Defected part of the cartilage was completely repaired with hyaline cartilage tissue within 24 weeks, even in the case of implantation of hydrogels without chondrocytes		
HEC/CS/GP hydrogel	A chitosan and chitosan-GP solutions were prepared separately	hBM-MSCs and rat BM-MSCs (rBM-MSCs) were resuspended in HEC, mixed with CS-GP and cultured up to 28 days	*In vitro*	Viability, proliferation, H&E, GAGs, proteoglycans staining	Naderi-Meshkin et al. (2014)
			HEC/CS/GP supported cell survival and proliferation of MSCs and differentiation towards cartilage-like tissue		
SF/HPMC/MA bioink	SF formed a presolution that could create a β-sheet; HPMC modified with methacrylate (MA) that could cross-link	BM-MSCs were mixed with SF/HPMC/MA to print ring shape scaffolds with a diameter of 8 mm, 8 layers, under UV radiation, followed by incubation to promote the formation of β-sheet structure	*In vitro*	Cell viability, gene expression (*COL1A2*, *ACAN, SOX9*)	Ni et al. (2020)
			BM-MSCs could survive and proliferate in the printed scaffold. Combining with growth factors, the genes further upregulated after chondrogenic differentiation		
MC/alginate bioink	Alginate mixed with MC. MC sterilized with autoclave, supercritical CO_2_ (scCO_2_), UV, and γ irradiation	Bovine primary chondrocytes (BPCs) isolated from metacarpal phalangeal joints were mixed with MC/alginate, bioprinted as a 3D constructs (9.5 mm × 9.5 mm × 1.4 mm per layer) and cultured up to 21 days	*In vitro*	Cell viability, DNA content, proteoglycans, nuclei, H&E staining	Hodder et al. (2019)
			Concerning cell survival and proteoglycan production, the rradiation and autoclaving were the best candidates for sterilization. The scCO_2_-treatment of MC resulted in an unfavourunfavorablesponse		
MC/alginate bioink	Bioinks contained plasmids encoding for RALA-pLUC or BMP2 or ca ombination of TGF-β3, BMP, 2 and SOX9 within networks of 3D printed fibers	Porcine BM-MSCs were encapsulated in MC/alginate bioink or 3D bioprinted to form cylindrical constructs and cultured for 4 weeks Acellular or MSC laden bioinks were used to print cylindrical dis, cs which were then implanted subcutaneously in nude mice up to 21 days	*In vitro* and *in vivo*	Cell viability, luciferase assay, the levels of BMP2 and TGF-β3, micro-computed tomography	Gonzalez-Fernandez et al. (2019)
			MC/alginate bioinks enabled the accelerated release of non-viral vector-pDNA complexes and enhanced the transfection of encapsulated MSCs. When used to deliver an appropriate combination of chondrogenic genes (*pTGF*-*pBMP*-*pSOX9*) to MSCs, these pore-forming bioinks were able to support robust and stable chondrogenesis of MSCs		
MC/alginate/HNT/PVDF bioink	Alginate mixed with MC, halloysite nanotube (HNT) and polyvinylidene fluoride (PVDF)	Human chondrocytes were seeded on 3D printed scaffolds and cultured up to 96 h	*In vitro*	Cell viability, GAGs	Roushangar Zineh et al. (2018)
			Cells attached, spread, and formed a polygonal structure with filopodia and were homogeneously distributed throughout the scaffold within the pores. By increasing the HNT in composition, living cell efficiency increased		
MC/alginate bioink combined with CPC	Alginate mixed with MC was used to create a biphasic scaffolds with calcium phosphate cement (CPC) gorming hydroxyapatite	hTERT-MSC cell line was mixed with bioink and 3D bioprinted into cuboid scaffolds (12 mm × 12 mm × 1 mm) with an alternating layer pattern (orientation of strands 0°/90°) and cultured up to 21 days	*In vitro*	Viability, cytoskeleton imaging	Ahlfeld et al. (2018)
			No change of viability within the MC/alginate strands of biphasic scaffolds was detected. Cells survived and proliferated along the CPC surface. Cells after migration to the CPC surface exhibited a spread, elongated morphology whereas cells inside the bioink maintained a roundish shape		
MC/alginate bioink combined with CPC	Alginate mixed with MC to create biphasic Scaffolds with CPC	Human chondrocytes from articular cartilage of femoral head of osteoarthritic patients were mixed with bioink and 3D bioprinted into square-shaped scaffolds consisting of 4 layers (total height 1.3 mm) and 7–8 mm of diameter after 1, 7, and 21 days of cultivation. Additionally, the MC/alginate-based bioink laden with hMSCs was combined with CPC	*In vitro*	Viability, proliferation, cytoskeleton aggrecan, collagen II staining, sGAGs, collagen II content, gene expression (*COL1A1*, *COL2A1*, *COL10A1*, *ACAN*, *SOX9*, *COMP*)	Kilian et al. (2020)
			Encapsulated cells survived and redifferentiated within MC/alginate in the absence and presence of a mineral CPC phase. Collagen type II, sGAGs were produced in monophasic and biphasic constructs. The mineralized zone as located in the calcified cartilage region was found to support chondrogenic ECM production by altering the ionic concentrations of calcium and phosphorus		
Electrospinning					
NaCS/gelatine scaffolds disks	NaCS incorporated into a fibrous gelatin construct	hBM-MSCs were grown in pellet cultures or seeded on NaCS/gelatine scaffolds up to 56 days	*In vitro*	Cell number, gene expression (*COL1A1*, *COL2A1*, *COL10A1*, *ACAN*, *CHAD*), collagen II, proteoglycans	Huang et al. (2018)
			Pellet cultures supplemented with NaCS expressed higher aggrecan and collagen type II gene expression than cultures without NaCS and had a chondrocyte-like morphology with intensive proteoglycans		
			MSCs on NaCS/gelatine scaffolds exhibited high extracellular ECM-associated gene expression		
pSC/gelatine scaffolds disks	pSC incorporated into a fibrous gelatin construct	hBM-MSCs were cultured on pSC/gelatine scaffolds up to 56 days	*In vitro*	Cell proliferation, cytoskeleton imaging, gene expression (*COL1A1*, *COL2A1*, *COL10A1*, *ACAN*, *SOX9, CHAD*), collagen II	Portocarrero Huang et al. (2017)
			Expression of chondrogenic markers was enhanced upon increasing pSC concentration in the scaffolds (up to 5%)		
pSC/gelatine scaffolds disks		hBM-MSCs were cultured on pSC/gelatine scaffolds up to 56 days	*In vitro*	Cell proliferation, sGAGs, collagen I, II, aggrecan, gene expression (*MMP2*, *COL1A1*, *COL2A1*, *COL10A1*, *ACAN*, *CHAD*, *SOX9*, *VEGF*)	Menezes and Arinzeh, (2020)
			MSCs undergoing chondrogenesis had higher expression of chondrogenic gene expression and a more homogenous cartilage-like matrix with the highest ratio of collagens type II to I on gelatin scaffolds containing pSC in comparison to native GAGs		
CAP scaffolds	Cellulose acetate phthalate (CAP) dissolved using various combinations of solvents	Chick embryo chondrocytes were seeded on gelatin-coated CAP scaffolds and cultured up to 12 days	*In vitro*	H&E staining	Shrestha et al. (2016)
			Attachment of the chondrocytes on CAP scaffolds prepared using dimethylformamide/tetrahydrofuran/acetone was confirmed		
Membranes					
Cellulose acetate membrane filter		Chondrocytes isolated from human articular cartilage of knee condyles we transferred to membranes and cultured for up to 21 days	*In vitro*	H&E staining, proteoglycans, collagen II, gene expression (*COL2A1*)	Mayer-Wagner et al. (2011)
			Membrane-based and pellet cultures showed the ability to promote redifferentiation of chondrocytes expanded in monolayer culture but membrane-based cultures have a short expression of *COL2A*		
PVA/CMC scaffolds	Polyvinyl alcohol (PVA) was mixed with CMC at different mass ratios. Porous scaffolds were created by the freeze-drying technique	Human articular chondrocytes were seeded on scaffolds and cultured for 7 days	*In vitro*	Cell viability	Namkaew et al. (2021)
			The incorporation of CMC modulated the pore architecture and increases the swelling ratio of scaffolds to promote cell adhesion without cytotoxicity		

**TABLE 3:** BNC applied to cartilage tissue engineering.

BNC type	System	Source of chondrocytes or stem cells and experimental design	Outcome	Analyses related to differentiation and proliferation	Reference
Pure BNC hydrogels					
BNC		Equine BM-MSCs were seeded on BC scaffold discs and cultured up to 14 days	*In vitro*	Cellular adhesion, viability and proliferation, SEM, GAGs	Favi et al. (2013)
			The BNC scaffolds were shown to be cytocompatible, supporting cellular adhesion and proliferation, and allowed for chondrogenic differentiation of MSCs		
BNC		The central defects of the bovine cartilage discs were filled with the BNC and, after embedding the constructs into the agarose, a culture medium was added. The constructs were kept for up to 8 weeks	*Ex vivo*	Aggrecan, collagen I, II, newly synthesized and cleaved collagen II, SEM, gene expression (ACAN, COL1A1, COL2A1), sGAGs	Pretzel et al. (2013)
			Cartilage discs displayed a preserved structural and functional integrity of the chondrocytes and surrounding matrix. Chondrocytes on the BNC surface showed signs of redifferentiation		
BNC		Rabbit BM-MSC were seeded on BNC discs and cultured up to 14 days	*In vitro*	Cell viability, SEM	Silva et al. (2018)
			BNC allowed the adhesion, expansion, and biointegration of BM-MSCs		
BNC (Xellulin^®^)		Human articular OA and non-OA chondrocytes were cultured in high density (HD) culture on a BNC for 7 days	*In vitro*	Proteoglycans, gene expression (*ACAN*, *S*OX9, COL1A1, COL2A1, MMP13)	Grigull et al. (2020)
			Chondrocytes kept the potential to express COL2A1 in all systems (pellet, HD hydrogel, and HD polystyrene). However, pellets did not develop degeneration and dedifferentiation markers		
Modified BNC					
Sulfated, phosphorylated and unmodified BNC membranes	Chemical sulfation with NH_2_SO_3_H and phosphorylation with H_3_PO_4_ of the BNC was performed to mimic GAGs of native cartilage	Primary bovine chondrocytes and human articular cartilage chondrocytes were seeded on BNC membranes of 15 and 8 mm diameter, respectively and cultured for 8 days	*In vitro*	Cell viability, adhesion morphology, gene expression (*COL1A1*, *COL2A1*)	Svensson et al. (2005)
			The chemical modifications did not enhance chondrocyte growth, while the material’s porosity supported viability and more extended morphology. Unmodified BNC supported viability of chondrocytes and did not induce the cells to differentiate into fibroblasts		
DHYL/DBNC/CS microcarriers	Nanofibrous microcarriers (NF-MCs) were prepared by crosslinking C2, 3-dialdehyde bacterial cellulose (DBNC), prepared by oxidation of BNC by NaIO_4_ solutions, with DL-allo-hydroxylysine (DHYL) and complexing CS with DHYL	BM-MSCs with NF-MCs were cultured under stationary conditions up to 7 days or GFP-labeled BM-MSCs with NF-MCs were cultured in a microgravity rotary system up to 21 days NF-MCs alone (selected with a pore size of 35 μm) or with GFP-labeled BM-MSCs were injected into rat femoral trochlear cartilage defect up to12 weeks	*In vitro*	Cell viability, GFP imaging, H&E, proteoglycans, collagen II, micro- computed tomography, gait analysis	Wang et al. (2018)
			BM-MSCs cultured in NF-MCs showed improved proliferation compared with those cultured in CS microcarriers. Under microgravity conditions, functional cartilage was generated		
			*In vivo*		
			The functional microtissue was used to repair a critical-size osteochondral defect successfully and was shown to be advantageous for tissue healing in a short time post-operation		
Modified BNC membrane	The artificial human auricle made of a BNC membrane, at least 25 mm thick, subjected to physical modification, including mold compression and placed in a bath with a high concentration of sodium hydroxide	BNC’s subcutaneous implantation resembles artificial human auricle in the back of rats for 14, 30, 90, or 720 days	*In vivo*	Macroscopic examination	Miśkiewicz et al. (2019)
			Resection after 14, 30, 90 and 720 days showed progression of the healing process and integration of the artificial skeleton into the animal body. There were no signs of change in the shape or structure of the skeleton		
Modified BNC membrane	Modified BNC produced from *N*-acetylglucosamine-fed cultures from metabolically engineered *K. xylinus* characterized by low crystallinity and lysozyme susceptibility	Human BM-MSCs were seeded on scaffolds (6 mm diameter discs) and cultured up to 4 weeks	*In vitro*	Cell viability, proliferation, SEM, sGAG content, gene expression (*ACAN*, *SOX9*, *COL2A1*), collagen II	Yadav et al. (2015)
			Porous scaffolds (pore sizes larger than the cells) supported hBM-MSCs attachment, proliferation and cartilage-specific matrix biosynthesis. The spatial cell arrangement and collagen type II and *ACAN* distribution resembled those in native articular cartilage tissue		
Densified BNC	BNC with increased cellulose content (17%) was produced, purified in a perfusion system, and compressed to increase the cellulose content	Densified BNC hydrogel disks (a diameter of 10 mm and height of 1 mm) were implanted intradermally on the back of the *Chinchilla* Bastard rabbits for 1 week	*In vivo*	H&E	Martínez Ávila et al. (2014)
			The biocompatibility tests demonstrated that densified BNC hydrogels are non-cytotoxic and cause a minimal foreign body response		
Air dried and freeze dried BNC	BNC was dried by air drying under ambient condition and freeze drying to produce BNC layers having different compressive moduli	Rat MSCs were seeded on BNC discs and cultured up to 28 days	*In vitro*	Cell viability, morphology, GAGs	Taokaew et al. (2014)
			MSCs attached and spread on the surface of the BNC. The softer freeze dried BNC promoted the differentiation of MSCs into chondrocytes. The differentiation was found to be difficult without using the differentiation agents		
BNC with relaxed fibers structure	BNC produced by genetically modified strain (*K. hansenii* ATCC 23769 *motAB* + strain)	ATDC5 cells were seeded on BNC discs and cultured up to 21 days	*In vitro*	Cell viability, morphology, GAGs	Jacek et al. (2018)
			*MotAB +* BNC with relaxed fiber structure supported ATDC5 cells growth to a higher extent than the wild-type BNC. ATDC5 cells seeded on *motAB +* BNC secreted the highest levels of GAGs		
Porous BNC	Scaffolds obtained by cultivating *K. xylinus* in the presence of agarose microparticles deposited on the surface of a growing BNC pellicle	Human primary chondrocytes were seeded on scaffolds (∼40 mm in diameter) and cultured up to 14 days	*In vitro*	Cell proliferation viability, cytoskeleton imaging, SEM	Yin et al. (2015)
			The introduction of pores with a size of 300–500 μm enabled chondrocytes to penetrate scaffolds, migrate and proliferate. Chondrocytes seeded on scaffolds displayed a 3D distribution extending throughout the porous BNC network		
Porous BNC	Porous BNC was produced by paraffin beads embedded during the fermentation process	Human primary chondrocytes derived from rseptorhino- and otoplasties were seeded on scaffold and cultured up to 14 days	*In vitro*	Adhesion, agreccan, collagen II, gene expression (*COL1A1*, *COL2A1*, *VCAN*)	Feldmann et al. (2013)
			Chondrocytes adhered to BNC, re-differentiated, produced cartilaginous matrix proteins and were located within the pores. Variations in pore sizes of 150–300 μm and 300–500 µm did not influence ECM synthesis		
Porous BNC scaffold	Porous BNC scaffolds were prepared by fermentation of *K. xylinus* in the presence of slightly fused wax particles with a diameter of 150–300 μm, which were then removed by extrusion	Neonatal articular chondrocytes from a cell line from the distal end of meta-carpal phalangeal bone cells were seeded onto rectangular-shaped scaffolds (0.25 cm^2^) and cultured up to 14 days. Chondrocytes from human cartilage were seeded on scaffolds and cultured up to 28 days	*In vitro*	SEM, confocal microscopy, GAGs, DNA content	Andersson et al. (2010)
			Cells tended to cluster within the pores, with cells only entering the pores at the surface layer. Cells produced ECM components in scaffolds when being arranged closely to each other		
Perforated BNC	BNC was scalpel-cut, laser-cut, or laser-perforated	Bovine and human articular chondrocytes were seeded on BNC samples up to 21 days	*In vitro*	Viability, H&E, GAGs, gene expression (*ACAN*, *COMP COL1A1*, *COL2A1*, *COL9A1*)	Ahrem et al. (2014)
			Unidirectionally perforated and in particular 3D-perforated BNC allowed ingrowth into and movement of chondrocytes throughout the BNC matrix. Cells displayed clear signs of re-differentiation		
Composites					
BNC/alginate bioink	BNC was disentangled into single cellulose nanofibrils by hydrolysis or aqueous counter collision (ACC) method that dissociates weaker intermolecular interaction, without chemical modification	hNCs mixed with ACC bioink and 3D bioprinted into the lattice-shaped constructs (5 × 5 × 1 mm with 1.2 mm spacing) were implanted into subcutaneous pockets on the backs of the nude mice up to 60 days	*In vivo*	GAGs, collagen II, FISH	Apelgren et al. (2019)
			Cell-laden structures were rapidly integrated, maintained structural integrity, and showed chondrocyte proliferation. Production of collagen II and GAGs was observed. FISH analysis confirmed that the GAG-positive cells in the 3D-bioprinted constructs were mainly of human origin		
BNC/κ-Car	BNC/κ-Car composites were prepared using *in situ* method with supplementation of the medium with κ-carragen (κ-Car)	ATDC5 cells were seeded on BNC discs and cultured up to 21 days	*In vitro*	Cell viability, gene expression (*COL1Α1*, *COL2Α1*, *RUNX2*, *SOX9*)	Cielecka et al. (2018)
			BNC/κ-Car composites supported the differentiation of ATDC5 cells to more chondrogenic phenotype and did not cause the chondrocyte hypertrophy		
GelMA/BNC	GelMA/BNC composite hydrogels were prepared by immersing BNC particles, obtained by homogenization of BNC membranes, in GelMA solution followed by photo-crosslinking	Human OA chondrocytes were suspended in GelMA solution and mixed with BNC suspensions and LAP. After UV light exposure cells were cultured up to 14 days	*In vitro*	Cell viability, collagen II, aggrecan, SOX9	Gu et al. (2021)
			Fluorescent stainings of SOX9, type II collagen and aggrecan were positive in most of the chondrocytes		
BNC/HA and BNC/GAG	Nanocomposites of BNC/HA were synthesized using simulated body fluid. BNC/GAG were prepared by incubation of BNC scaffolds with chondroitin sulfate sodium salt	Human articular chondrocytes or human ATSCs were seeded on scaffolds up to 28 days Scaffolds were inserted into a rat subcutaneous pocket created in the region corresponding to the lower thoracic vertebrae up to 45 days Bilayer scaffold (BNC/HA and BNC/GAG placed one over the other) were implanted into rat osteochondral defect created in the patellar groove of the knee joints for 90 days	*In vitro*	Cell viability, cytoskeleton imaging, sGAGs, proteoglycans, micro- computed tomography	Kumbhar et al. (2017)
			Higher proliferation and synthesis of sGAGs by chondrocytes and ATSCs was observed of on BNC/GAG scaffolds		
			*In vivo*		
			The scaffolds allowed tissue ingrowth with no inflammation or immunological reactions. Implantation of acellular bilayered scaffolds in osteochondral defect simultaneously accelerated the regeneration of articular cartilage and subchondral bone. Scaffolds displayed excellent biodegradative resorption properties		
Bilayer BNC scaffolds	Bilayer scaffold (densified BNC layer and BNC/alginate porous layer) was produced with ionic liquid used to attach the layers	hNCs were seeded on BNC scaffolds and cultured for up to 6 weeks hNCs and bone marrow mononuclear cells encapsulated in alginate were seeded in bilayer BNC scaffolds and implanted subcutaneously on the dorsal side of nude mice for 8 weeks	*In vitro*	GAGs, aggrecan, collagen I, II, gene expression (ACAN, VCAN, COL2A1, COL1A1), proteoglycans	Martínez Ávila et al. (2015)
	BNC disks with cellulose content of 17% were produced by compression, whereas BNC/alginate composite scaffolds were fabricated by a freeze-drying		Bilayer BNC scaffolds supported the redifferentiation of NCs to a more chondrogenic phenotype, which led to neocartilage formation. The high porosity layer (75% porosity with a mean pore size of 50 ± 25 μm) supported the ingrowth and homogeneous distribution of NCs		
			*In vivo*		
			Deposition of cartilage matrix components were observed in constructs. The results showed the formation of neocartilage in the porous layer		
BNC double-network hydrogel	The bilayer structure with two different hydrogel layers (PGA alginate) with incorporated BNC was synthesized via a three-step cross-linking procedure. Additionally, hydroxyapatite particles with two different sizes were introduced into the bilayer hydrogels, for promoting cartilage matrix deposition and for enhancing compression modulus and osteogenesis	Hydrogels were implanted into a cylindrical osteochondral defect in the rabbit medial femoral condyle for 12 weeks	*In vivo*	H&E, micro- computed tomography	Zhu et al. (2018)
			The newborn hyaline cartilage with smooth surface and the subchondral bone reconstruction was observed at 12 weeks postoperation, which is much faster than the reported similar bilayer hydrogel scaffold without hydroxyapatite		

## Plant-Derived NC-Based Scaffolds for Cartilage Tissue Engineering

The use of NFC to improve the physical properties of hydrogels has sparked a lot of interest in recent years. NFC is the smallest fibrous component of cellulose fibers with diameters ranging from 5 to 60 nm depending on the origin. NFC has been deemed a top choice for various applications in the material science industry due to its abundance, high surface area, water retention value, transparency, and sustainability. Compared to CNC, NFC has a higher aspect ratio and can form robust physical entanglements and networks in composites. These characteristics make the gels far more durable than when the network is created solely by weak hydrogen bonds between water and fibrils. Additionally, NFC has a substantially lower Youngs modulus in tension (around 30 GPa) than CNC, which varies depending on the delamination procedure (Tanpichai et al., 2012). The biocompatible, injectable, mechanically stable, and slowly degradable NFC/chitosan (CS) thermosensitive hydrogel was shown to be ideal for cartilage regeneration. When CS and β-glycerophosphate (GP) are combined in aqueous solution, stimulus-responsive *in situ* forming gels are formed due to a combination of pH- and temperature-dependent interactions. However, the use of CS/GP hydrogels is limited due to poor mechanical strength, rapid deterioration, and loss of spatial support, whereas combining NC and CS has been shown to increase the latter’s biocompatibility, biodegradation, and mechanical characteristics. The chondrogenesis ability of human dental pulp stem and progenitor cells (hDPSCs) embedded in NFC/CS/GP hydrogel was validated by histological and immunohistochemical studies as well as gene expression, suggesting that NFC/CS/GP composite hydrogels can serve as a promising stem cell-based cartilage repair approach that is minimally invasive. *In vivo* studies demonstrated that 3D cell NFC/CS/GP constructs elicited an immunological response that had a favorable impact on the scaffold microenvironment. The immune response manifested itself as an acute inflammatory reaction which attracted macrophages. Macrophages together with hDPSCs secreted cytokines and growth factors necessary for the regeneraton of injured tissue (Talaat et al., 2020).

A combination of crosslinked sodium alginate and NC for cartilage tissue engineering has been investigated thoroughly. Sodium alginate offers structural stability in these composite hydrogels through chemical crosslinking, which aids in transforming the hydrogel into a solid substance. Calcium ions (crosslinker) can be added to sodium alginate to replace the sodium ions, forming strong bonds between alginate chains. The concentration of a crosslinker controls characteristics of the hydrogel by adjusting the structural and mechanical properties of the solid material (Lin et al., 2011). It was shown that increased CaCl_2_ concentration changes the overall architecture, pore size, and porosity. CaCl_2_ concentration appeared to impact CNC more than other NC types (NFC or a blend of CNC and NFC). Depending on the NC form, the hydrogels had 34–50% porosity with average pore diameters ranging from 0.22 to 0.91 µm (Al-Sabah et al., 2019).

Because of the low cytotoxicity and structural similarity with ECM, NFC with alginate in the form of hydrogels attracts attention in bioink formulation for 3D printing. 3D printing is a promising field that aspires to generate functional tissue-like constructs with precision and adaptability for tissue/organ regeneration replacement. 3D printing is an additive manufacturing technique in which a layer-by-layer computer-controlled deposition process creates 3D models. Construction of biological structures with tissue-specific geometry is now possible thanks to 3D printing and its ability to mimic the heterogeneous and complicated native tissues. This method allows hydrogels to be dispensed in three dimensions with increasing precision and resolution. Cells are enclosed in a uniform density and quantity within the printed gel in contrast to standard two-step methods involving inoculating cells into pre-fabricated scaffolds. Cell distribution is often diverse in the latter approach and the biomaterial may not be fully colonized. 3D printing is classified into numerous types based on the operating mechanism, the raw material, the energy sources, and the biological tasks. Inkjet-based, laser-based, and extrusion-based printing are the three basic techniques used to classify 3D printing. Extrusion printing is the most extensively used procedure in TE for producing cell-laden hydrogels, owing to its well-known benefits such as a low cost, ease of software or hardware upgrades, and excellent cellular viability (Martínez Ávila et al., 2016; Curvello et al., 2019).

So far, no proof of pure NC hydrogels being used for 3D bioprinting has been found. Shear thinning is much improved when NC is combined with other polymers such as alginate. Alginate increases gel viscosity by acting as a crosslinker. It strengthens the scaffold and ensures that the shape is maintained throughout the procedure. Because of its excellent printing accuracy, which can be attributed to its dominant elastic behavior, NC as a primary component in a bioink might improve shape fidelity. The addition of NC to bioinks in concentrations ranging from 2 to 10% provides better shear thinning, mechanical strength, and water retention (Toit et al., 2020). Gatenholm *et al.*, for the first time, proposed NC for producing implants and scaffolds for tissue engineering applications utilizing inkjet printing in 2010 (Gatenholm et al., 2010).

Markstedt *et al.* developed a biobased ink comprised of crosslinked 2.5% (w/v) NFC, 10–40% alginate, and CaCl_2_. NFC, produced from the bleached sulfite softwood cellulose pulp consisting of 40% pine (*Pinus sylvestris*) and 60% spruce (*Picea abies*) by combined refining, enzymatic treatment, and high-pressure homogenization, was mixed with alginate (150–250 kDa). They tested NFC/alginate compositions (90/10, 80/20, 70/30, 60/40 w/v%) and 80/20 appeared to be the best ink formulation based on all the outcomes, including rheological characteristics, compressive stiffness, and shape deformation after ionic crosslinking with Ca^2+^. The ink exhibited good gelling during crosslinking and minimum shape deformation and was demonstrated to be a viable host for human nasoseptal chondrocytes (hNCs) that were encapsulated and 3D bioprinted into gridded constructs (Markstedt et al., 2015). 60/40 NFC/alginate composition with human induced pluripotent stem cells has shown promising results to stimulate cartilage formation as well (Nguyen et al., 2017).

CELLINK AB (Gothenburg, Sweden) sells a bioink containing NFC/alginate (2/0.5 w/w%) under the CELLINK^®^ trademark. On a commercial level, CELLINK^®^ offers various ready-to-use bioinks for a variety of tissue engineering applications. CELLINK^®^ has a high level of consistency, printability and compatibility with a wide range of cell types. This bioink series has been extensively examined in both *in vitro* and *in vivo* research. It was shown to support chondrocyte proliferation and differentiation with excellent viability and cartilage ECM formation. CELLINK^®^ was used to bioprint human nasal chondrocyte-laden patient-specific auricular constructions with an open inner structure and good shape fidelity. hNCs proliferated and underwent chondrogenesis, resulting in the formation of cartilage-specific ECM components (Martínez Ávila et al., 2016). Additionally, 3D bioprinted constructs were produced using CELLINK^®^ with hBM-MSCs alone (Apelgren et al., 2017; Möller et al., 2017) or with hBM-MSCs in combination along with hNCs (Apelgren et al., 2017; Möller et al., 2017; Apelgren et al., 2018). A further proliferative impact involving chondrocyte synthesis of GAGs and type II collagen was reported in constructions containing a mixture of chondrocytes and stem cells (Möller et al., 2017).

The long-term (10-months study) outcomes on chondrogenesis associated with transplanted 3D-bioprinted CELLINK^®^ demonstated no adverse events, such as ossification, neoplasms, or necrosis (Apelgren et al., 2021). Also, a sulfated version of alginate that can bind FGF, TGF, and hepatocyte growth factors (HGF) was combined with NFC to form a printable bioink with better chondrogenic characteristics compared to inertia alginate-containing bioinks (Müller et al., 2017).

CNC can also be used in nanocomposite bioinks because of its intrinsic features like high mechanical strength, good biocompatibility, high surface area, and simplicity of surface modification. Depending on the source, the crystalline percentage can range from 50 to 90%. The elastic moduli of the CNCs were determined to be around 105–168 GPa (Kuhnt and Camarero-Espinosa, 2021). Due to reversible hydrogen bonding, CNCs were introduced in covalently crosslinked hydrogels to improve mechanical strength and provide viscoelastic behavior. Mainly, alginate mixed with CNC as an additive has emerged as a promising bioink for fabricating artificial tissues (Jessop et al., 2019). Besides, to increase the mechanical properties of the structures, a hybrid bioink was created by mixing CNCs and a gelatin methacryloyl/hyaluronic acid methacrylate (GelMA/HAMA) solution. CNC-GelMA/HAMA was used as the structural component and GelMA/HAMA as the cytogel containing ATDC5 cells. GelMA is a photoreactive gelatin derivative possessing Arg-Gly-Asp tripeptide that promotes cellular attachment, spreading, and differentiation, as well as matrix metalloproteinase sequences (Kurian et al., 2021). The reinforced bioinks demonstrated good printability, shear-thinning characteristics, structural support and structural integration in 3D bioprinted constructions. Meantime, the soft bioinks in the GelMA/HAMA mixture offered a favorable milieu for cell multiplication. As a result, ATDC5, a mouse chondrogenic cell line, stayed alive during the bioprinting process (Fan et al., 2020).

To improve the mechanical properties, CNC was also introduced into polycaprolactone (PCL)/poly (acrylic acid) (PAA) hydrogel. PCL, a biodegradable and biocompatible polyester approved by the Food and Drug Administration, has gained a lot of scientific interest as an implanted biomaterial. Despite having the essential toughness and mechanical qualities, PCL is hydrophobic, resulting in insufficient cell attachment. Semi-interpenetrating polymer networks (semi-IPNs) were incorporated to restore the system’s hydrophilicity to address this problem. Semi-IPNs are composite polymeric materials made by synthesizing or crosslinking a polymer network in the presence of non-crosslinked polymers. When PCL is combined with hydrophilic polymers, it can provide excellent hydrophilicity while maintaining mechanical strength. Therefore, PCL and CNC were caught in a network of acrylic acid. The mechanical qualities of artificial cartilage were improved using an optimal amount of CNC, which was 0.5%. The semi-IPNs’ biocompatibility was proven by testing viability and cell attachment (Pourbashir et al., 2020).

Surface-modified CNCs have also been examined as green and biocompatible collagen hydrogel reinforcing agents. Compared to CNC/collagen hydrogels without Schiff base links, a nanocomposite aldehyde-functionalized CNC (a-CNC)/collagen hydrogel crosslinked by dynamic Schiff base bonds displayed quick shear-thinning, self-healing, and enhanced elastic modulus. After extrusion *in vitro*, BM-MSCs encapsulated in the a-CNC/collagen hydrogel showed good cell viability. MSCs encapsulated in the a-CNC/collagen hydrogel and injected subcutaneously demonstrated better implant integrity and cell retention. *In vivo* subcutaneous injection showed that a-CNCs/collagen hydrogel acted as a protective biomaterial of BM-MSCs by minimally invasive procedures. Therefore, the hydrogel would not only protect cells during injection, but also fit into the cartilage defect, making MSC delivery for cartilage regeneration a viable option (Zhang et al., 2020).

Sulfated CNC (S-CNC) was employed to create a porous multilayer scaffold made up of an anisotropic poly (d,l-lactide) (PLA) superficial layer with tubular pores oriented parallel to the subchondral bone, an isotropic middle layer made up of PLA and sulfated CNCs, and a deep anisotropic layer made up of PLA and phosphated CNC (P-CNC) with tubular pores oriented orthogonal to the subchondral bone. These scaffolds spatially controlled the morphology, orientation, and differentiation of human chondrocytes. The constructs enabled the growth of tissue with characteristics similar to the natural equivalent and encouraged localized hydroxyapatite production to allow integration with the subchondral bone (Camarero-Espinosa et al., 2016).

Another interesting approach with CNC-based hydrogels provides a new methodology to non-invasively monitor biomaterial behavior after implantation. The ability to image hydrogels is helpful for both confirming that the hydrogel was successfully delivered to the target region and monitoring the erosion of the hydrogel over time. Chen *et al.* developed a functional, visualizable ultrasmall superparamagnetic iron oxide (USPIO)-labeled natural hydrogel system for semi-quantitative monitoring of cartilage deterioration and revealed hyaline cartilage regeneration using multiparametric magnetic resonance imaging (MRI). A silk fibroin (SF) was introduced into the CNC scaffold forming CNC/SF blend hydrogel to improve cartilage regeneration. MRI showed that the USPIO-labeled hydrogel had enough contrast to track the degradation process. As a result, this approach gives helpful information for noninvasive monitoring and therapeutic efficacy of implanted hydrogels (Chen et al., 2018). Yang *et al.* also created a type of USPIO-based hydrogel for cartilage regeneration that encouraged the differentiation of BM-MSCs into chondrocytes by mixing USPIO-KGN with CNC/Dex (dextran). KGN was discovered to be produced over an extended period, attracting endogenous host cells and encouraging BM-MSC differentiation into chondrocytes for efficient cartilage regeneration, as evidenced by both *in vitro* and *in vivo* investigations (Yang et al., 2019a).

Finding suitable solvents is the most challenging part of preparing NC-based hydrogels since NC is insoluble in water and forms a colloidal suspension. Therefore, many chemical processes and modifications have been established to dissolve cellulose in water or organic solvents. Recently, a novel class of cellulose solvent, aqueous lithium bromide (LiBr) was used to form cellulose hydrogels with various shapes from a rigid cylinder to soft sponges from ashless filter paper pulp. Additionally, NaCl particles were distributed in cellulose solution. Finally, all NaCl particles were rinsed out following gelation, resulting in a soft sponge-like cellulose hydrogel structure with micro-pores of about 300 μm in diameter connected by 100-μm pores. Rabbit chondrocytes proliferated and differentiated into cartilage tissue when seeded on such a hydrogel (Isobe et al., 2018).

To sum up, plant-derived NC holds promise for cartilage tissue engineering. Plant NC-based nanomaterials can be produced in the form of CNFs or CNCs. Chemical, enzymatic, and mechanical treatments, as well as the combination of these methods, can be applied to produce NC with desired properties. The surface of CNF and CNC with functional groups allow their use for the implementation of cross-linking chemistry in the hydrogel (Xu et al., 2018). An overview of plant-based scaffolds used for studies on cartilage tissue engineering with chondrocytes and stem cells is listed in Table 1.

## Cellulose Derivatives as Scaffolds for Cartilage Tissue Engineering

**FIGURE 5:**
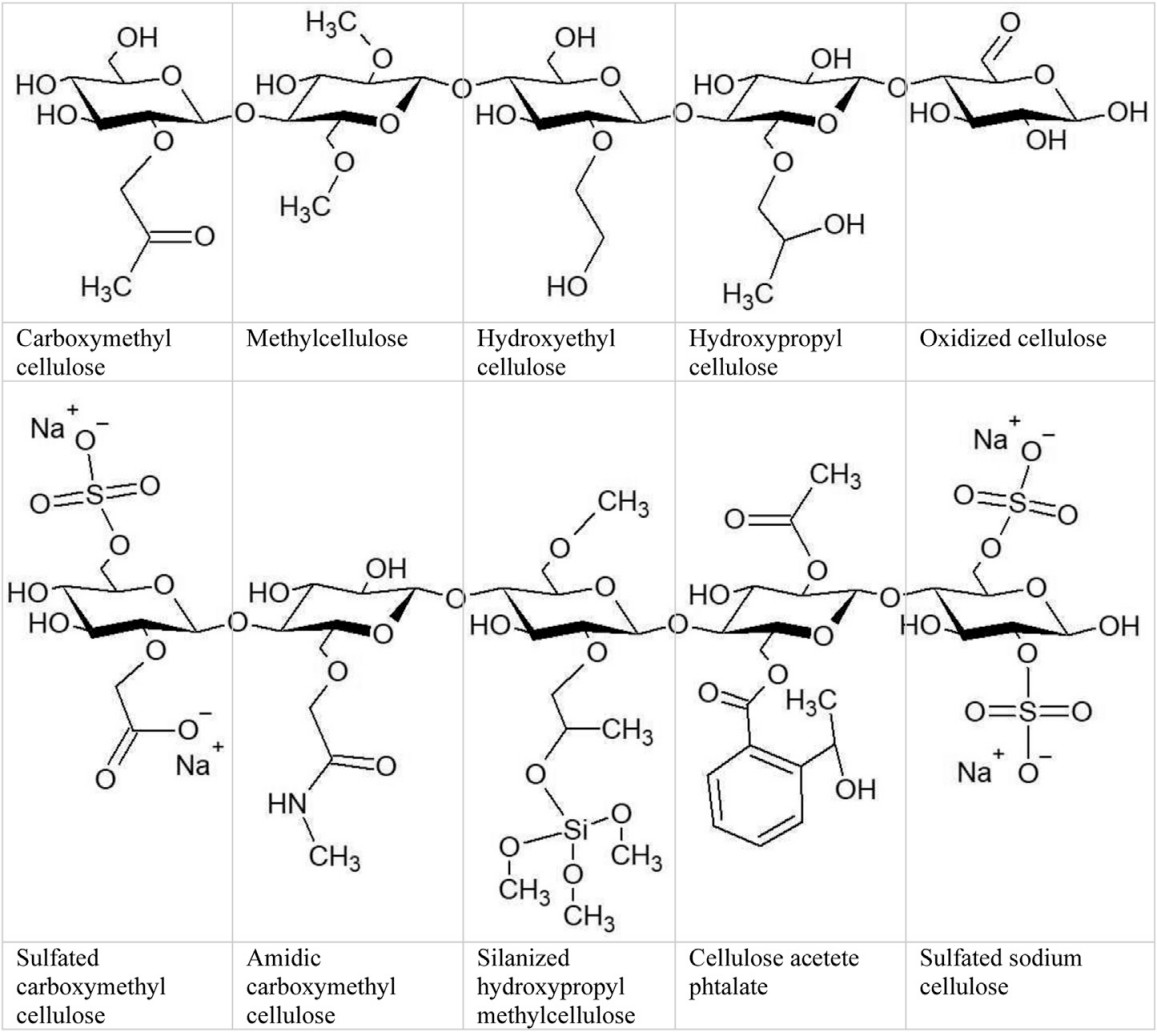
Cellulose and its derivatives. Hydroxyl groups connected with carbon 2, 3 and/or 6 allows to formation esters or ethers bonds in variable ratio.

The most important cellulose derivatives, cellulose ethers and esters, have been commercially available for many years. Cellulose ethers made by replacing the hydroxyl groups in cellulose with methyl, hydroxyethyl, hydroxypropyl, and other similar groups are soluble in alkaline aqueous phases and several organic solvents. The esterification of cellulose allows it to be processed into various forms, including solutions, fibers, and 3D structures. Cellulose acetate, cellulose acetate propionate, cellulose acetate butyrate, and other cellulose esters are widely used (Seddiqi et al., 2021). Physical and chemical crosslinking has been utilized to build cellulose-based hydrogels from MC, CMC, HPMC, hydroxypropyl cellulose (HPC), or cellulose acetate (CA) (Table 2; Figure 5).

The cellulose ether CMC is the most widely used. Enzymatically crosslinked injectable hydrogel consisting of CMC, sulfated CMC (sCMC), gelatin was prepared for delivery of a combination of caprine articular chondrocytes (ACs) and infrapatellar fat pad derived mesenchymal stem cells (IFP-MSCs) to a cartilage lesion site to induce chondrogenesis (Arora et al., 2017). Oxidized CMC/gelatin (Balakrishnan et al., 2013) and amidic derivative of CMC (CMCA) hydrogels are also examples of injectable, biodegradable and ECM mimicking hydrogels supporting growth and differentiation of chondrocytes that integrate well inside the cartilage defect area, stabilizing and preventing further deterioration (Leone et al., 2008).

MC, categorized as a lower critical solution temperature polymer because of its unique ability to generate thermo-reversible hydrogels when heated, was used to create polyurethane (PU)/MC composite scaffolds. In a custom-built bioreactor, PU/MC composites with hBM-MSCs were subjected to a mix of compression and shear pressures. Mechanical stimulation increased chondrogenic gene expression while histological investigation revealed sulfated GAGs and collagen II only in loaded specimens, indicating that MC hydrogels are suitable for load-induced MSC chondrogenesis. The use of MC as a thickening additive for alginate extrusion-based 3D printing is a new technique (Schütz et al., 2017) showing great promise in the bioplotting of several cell types, including chondrocytes and MSCs (Gonzalez-Fernandez et al., 2019; Hodder et al., 2019). MC was also employed to create hierarchical scaffolds to reproduce the zonal organization of articular cartilage. Encapsulated cells underwent redifferentiation. The presence of a mineralized zone supported chondrogenic ECM production by altering the ionic concentrations of calcium and phosphorus (Kilian et al., 2020).

Silanized-hydroxypropyl methylcellulose (Si-HPMC) is a more complex cellulose derivative used to fabricate hydrogel scaffold materials in cartilage tissue engineering to promote the proliferation and differentiation of cells. In a crosslinking procedure involving the condensation of silanol groups, Si-HPMC is steam sterilizable, injectable, and self-sets at physiological pH. This method of crosslinking without the use of an exogenous reticulating agent is typically associated with high biocompatibility. Once reticulated, this cellulose-based hydrogel has only 2% dry polymer and 98% water, closely mimicking the high hydration of articular cartilage ECM. Although Si-HPMC’s mechanical properties (compressive modulus approximately 2.9 kPa) differ from those of articular cartilage (around 30 kPa), it provides a 3D environment in which cells can proliferate and create cartilage ECM (Rederstorff et al., 2017). Si-HPMC is a self-crosslinking hydrogel used for the 3D culture of chondrocytes and MSC (Vinatier et al., 2005; Vinatier et al., 2009; Merceron et al., 2011). It has also been preclinically investigated to repair stiffer tissues like cartilage, with less promising results (Vinatier et al., 2007). Also, composite of Si-HPMC with nano-reinforcement clay known as laponites (Boyer et al., 2018), GY785 (a high-molecular weight marine exopolysaccharide) or and Si-chitosan (Boyer et al., 2020) were synthesized and demonstrated to be helpful in treating cartilage defects. Interestingly, in the canine osteochondral defect model, defects filled with Si-HPMC with or without adipose stromal cells, revealed a significant osteochondral regeneration (Boyer et al., 2020).

HEC (hydroxyethylcellulose) can serve as a cross-linking to form HEC/CS/GP hydrogels since chitosan can undergo sol-gel transition at 37°C when combined with GP and HEC. Apart from imitating the GAG structure, HEC/CS/GP hydrogels promote MSC survival and proliferation and chondrogenic differentiation of encapsulated chondrocytes and MSCs. As a result, this form of hydrogel may be used to patch cartilage defects during arthroscopic surgeries (Naderi-Meshkin et al., 2014).

Chemically sulfated cellulose may also serve as a scaffold for cartilage tissue engineering. Sulfation of cellulose improves solubility with encapsulated MSCs disrupting intermolecular hydrogen bonds, potentially extending its applicability to a broader range of tissue engineering applications. Huang *et al.* investigated a completely sulfated version of sodium cellulose (NaCS), which resulted in considerable collagen II and aggrecan upregulation by hBM-MSCs. Electrospinning was also used to create scaffolds containing NaCS and gelatin. The lowest concentration of NaCS (0.1%) resulted in the higher synthesis of collagen type II. Increased NaCS concentrations may hinder chondrogenesis because of irreversible growth factor-biomaterial binding (Huang et al., 2018). The same authors also used partially sulfated cellulose (pSC) in gelatin hydrogels. They found that increasing the pSC concentration in the scaffolds upregulated the expression of chondrogenic markers, indicating that pSC could be used as a scaffolding material for cartilage tissue engineering (Portocarrero Huang et al., 2017; Menezes and Arinzeh, 2020).

To overcome the limitations of native cellulose, chemical modifications are necessary. The fabrication of scaffolds using cellulose derivatives, i.e., MC, CMC, HPMC, HPC, or CA has been demonstrated with the aim of cartilage repair. The biomechanical and physicochemical features of cellulose derivatives depend on the nature of introduced chemical group and degree of substitution. Manipulating the concentrations of the solvent-soluble derivatives of cellulose can alter the stiffness of the formed hydrogels (Chinta et al., 2021). An overview of cellulose derivatives applied to cartilage tissue engineering is listed in Table 2.

## BNC-Based Scaffolds for Cartilage Tissue Engineering

**FIGURE 6:**
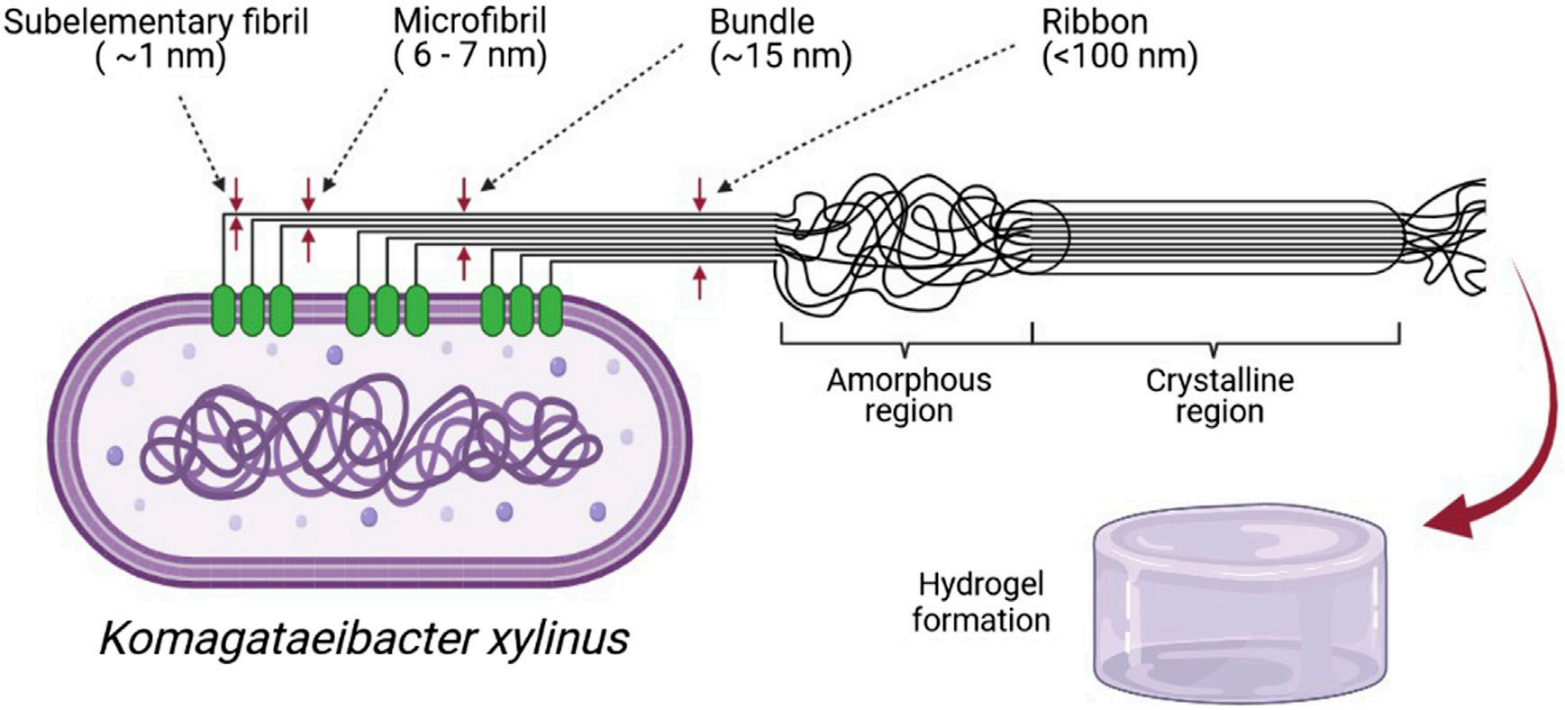
Bacterial cellulose is produced by cellulose synthases found in groups in the cell wall and (Lee et al., 2014). Cellulose is pulled outside the cell and sub-elementary fibril is formed. During cell movement, extracted cellulose connect and create microfibrils, bundles, and ribbons. BNC, besides high purity, is characterized by a high crystalline region (80–90%) (Lee et al., 2014), a part with high stacked polysaccharide chains. *Komagateibacter* during stationary culture produce BNC in a form of hydrogel (created in BioRender.com).

BNC-based hydrogels (Figure 6) are particularly interesting materials for cartilage tissue engineering because of their high purity (lack of hemicelluloses or lignins). In addition, BNC is biocompatible, has strong mechanical properties, a high water binding capacity, and a large surface area with many hydroxyl groups. Besides, 3D nanofibrous network of BNC resembles ECM.

But the lack of biodegradability of BNC, like with NFC or CNC, is a serious disadvantage. However, methods to overcome this constraint have been developed, such as using a metabolically engineered K. xylinus strain to produce lysozyme-modified degradable BNC (Yadav et al., 2010) or electron beam irradiation before implantation (An et al., 2017). Biodegradative resorption was also observed in the case of BNC-hydroxyapatite (BNC/HA) and BNC/GAG nanocomposite bilayered scaffolds that mimic bone and cartilage, respectively (Kumbhar et al., 2017).

Another limitation of employing BNC for cartilage tissue engineering applications is the pore size of BNC (diameter of ∼0.02–10 μm depending on the bacterial strain and the culture conditions) that is smaller than the diameters of mammalian cells. This feature prevents cells from migration across the scaffold. The upper surface of BNC (in contact with air) has a dense architecture with much smaller pores (below 2 μm), whereas the lower surface (in contact with the culture liquid) has pores with a much bigger diameter (Ahrem et al., 2014). Cell attachment, migration, and ingrowth are all influenced by the scaffold’s pore size. Large pores in the scaffold allow efficient delivery of nutrients, gas diffusion, and metabolic waste disposal. Still, they can also contribute to poor cell attachment and intracellular signaling, whereas tiny pores can have the opposite effect. Chondrocytes were shown to prefer scaffolds with pore sizes ranging from 250 to 500 μm for improved proliferation and ECM synthesis. The growth rate, the amount of GAG secretion, and the expressions of the differentiation gene markers increased as the pore size became larger. On the other hand, the cells growing on scaffolds with the smaller pores were frequently found to be dedifferentiated (Lien et al., 2009).

Several methods have attempted to create porous BNC scaffolds, *e.g*., cultivating *K. xylinus* with porogen materials (Bäckdahl et al., 2008). When porogen/particle-leaching approaches are employed, the porogen particles are removed from the system by a leaching process, leaving a highly porous polymer scaffold. Paraffin wax particles of 150–300 μm in diameter were used as a porogen to form a porous BNC material evaluated as a scaffold for cartilage regeneration. Human chondrocytes entered the pores to some extent and synthetized ECM components. However, removing wax from BNC and controlling the size of pores are issues to deal with. Besides, even though human chondrocytes tolerated the sponge-like material created with this method, penetration of these cells into the scaffold was limited to the outermost layers (Andersson et al., 2010). BNC porosity generated by paraffin beads was also demonstrated to augment a chondrogenic phenotype of nasal and auricular chondrocytes (Feldmann et al., 2013). Another example of porous BNC scaffolds formation includes cultivating *K. xylinus* in the presence of agarose microparticles. Using a microfluidic method, monodispersed agarose microparticles with a diameter of 300–500 μm were deposited over synthetized BNC pellicles and integrated into the polymer when *K. xylinus* cells migrated upward through the growing pellicle. As a result, human chondrocytes divided, infiltrated the 3D porous network, were uniformly distributed, and had better viability compared to unmodified BNC (Yin et al., 2015). Using bilayer BNC composites (micro-porous layer created by freeze drying with alginate beads) implanted subcutaneously into mice confirmed that such composites have good mechanical stability, preserve structural integrity, and enable cell ingrowth. *For in vivo* studies bilayer BNC scaffolds were seeded with a low number of freshly isolated human chondrocytes combined with freshly isolated mononuclear cells. After 8 weeks chondrocytes showed the deposition of proteoglycans and type II collagen (Martínez Ávila et al., 2015). Other BNC porous composites containing GAGs placed on the surface of the cellulose membrane (BNC/GAG) were found to promote chondrogenesis and facilitate infiltration of host tissue, resulting in a well-spread blood vascular network at the implantation site (Kumbhar et al., 2017).

Laser perforation by post-processing step is another technology that has shown promise to modify BNC surface morphology. Generally, laser treatments create parallel-channel arrays of pores (Jing et al., 2013). Ahrem *et al.* demonstrated the possibility of 3D laser perforation of BNC hydrogels with a pulsed CO_2_ laser system which generated round-shaped channels of specified arrangement. The 3D-perforated BNC had high biocompatibility, and the resultant channels allowed chondrocytes to migrate into the BNC, produce matrix, and maintain their phenotypic stability (Ahrem et al., 2014).

Genetic modifications of BNC-producing strains are the next option. BNC with relaxed fiber structure was obtained by overexpression of *motA* and *motB* genes in *K. hansenii* ATCC 23769. MotA and motB form a proton pump and are thought to be involved in cell motility. Their overexpression (motA+, motB+, and motAB+) resulted in bacterial cell elongation (or filament production) and increased colony-spreading abilities, making BNC more porous and relaxed. The *K. hansenii* mutant-derived BNC appeared to be potential support for the growth of chondrogenic cells and encouraged their chondrogenic-like activity (Jacek et al., 2018).

Because BNC and plant-derived NC have similar chemical compositions, BNC can be modified using the same chemical procedures, namely phosphorylation, carboxylation, or acetylation. Chemical sulfation and phosphorylation of BNC can add a charge and mimic GAGs of native cartilage. However, in this particular case, those modifications did not affect chondrocyte growth (Svensson et al., 2005). Other studies have shown that composite hydrogels with BNC can also be utilized to support differentiation and growth of chondrocytes and MSCs. BNC with a 17% increase in cellulose concentration was comparable to auricular cartilage in terms of mechanical strength and host tissue reaction. The implants were strongly attached to the surrounding soft tissue after 1 week of retention in the body, while blood and tissue fluid were absorbed into the implant material (Martínez Ávila et al., 2014).

Because of its unique physiochemical characteristics and outstanding biocompatibility, BNC has emerged as a viable biomaterial for cartilage tissue engineering applications. The simplicity with which it may be adjusted to suit any desired direction by chemical reactions, structural modifications, or the incorporation of active components into the BNC structure has made it an excellent biomaterial for preparing scaffolds. To improve issues connected with biodegradability and porosity, enzymatic chemical, and genetic engineering technologies could be used. An overview of BNC applied to cartilage tissue engineering is listed in Table 3.

## Conclusion

Repairing cartilage tissue remains a serious clinical challenge. The global prevalence of cartilage problems has risen rapidly in recent years, and it is expected to quadruple by 2040 (Stampoultzis et al., 2021). With the rapid breakthroughs in tissue engineering, cartilage regeneration *via* transplantation of artificial constructs has become a viable approach. However, despite promising results *in vitro*, the lack of artificial constructs that mimic the native tissue’s biomechanical and biochemical milieu has hampered its application in clinical practice.

Very challenging aspects of cartilage TE are related to selecting the cell source together with cellular differentiation and expansion. Chondrocytes appear to be the most promising starting material that promises the regeneration and healing of cartilage. Even though chondrocytes seem to be the primary choice, difficulties such as low proliferation rate and dedifferentiation are likely. Due to restricted sources and challenging collection processes, differentiated chondrocytes are difficult to obtain in appropriate amounts. As a result, other cell sources have been investigated, and the use of mesenchymal stem cells is an effective and safe technique to stimulate chondrogenesis. Therefore, an optimal scaffold would be one that can maintain a balance between chondrocyte proliferation and MSC differentiation within a 3D matrix (Grigolo et al., 2011).

Nanocellulose offers excellent potential in tissue engineering because of its biocompatibility, non-toxicity, water holding capacity, and superior mechanical properties. Exploitation of plant-derived biopolymers in TE is a new sector with untapped promise for developing more advanced functional materials from the world’s most plentiful and sustainable resources, as well as responding to global trends toward customized medicine and therapy (Xu et al., 2018). Nanocellulose is now widely used as part of a new generation of nanomaterials for versatile global biomedical applications due to the promotion of cellular interactions and tissue development mimicking the extracellular matrix. Although currently NC application in cartilage tissue engineering is mainly at the laboratory stage, in the future, personalized implants can be manufactured using 3D printing technology. NFC, in particular, meets the application criteria for bioplotting, inkjet printing, and extrusion-based printing due to its inherent gel-formability (Markstedt et al., 2015). Also, BNC is a unique functional material that has already demonstrated considerable promise for biological applications in its natural state (Pretzel et al., 2013) or as a component of bioinks (Apelgren et al., 2019). Furthermore, its ability to be modified and composited underlines its suitable position in cartilage tissue engineering (Svensson et al., 2005).

However, obstacles must be overcome before NC-based biomaterials will be used in therapeutic settings. The safety related to long-term stability of the regenerated cartilage is an important issue. NC degrades slowly in the human body due to the lack of enzymes that break β-1,4 glycosidic bonds. Also, a high degree of crystallinity may contribute to its nonbiodegradability. Therefore, many attempts have been made to improve the degradability of cellulose products *in vivo* (*e.g*., Yadav et al., 2010; An et al., 2017). On the other hand, nonbiodegradable NC could be employed as a long-lasting support material in applications such as cartilage meniscus implants.

Another essential difficulty restricting nanocellulose’s use is its pore size. Scaffolds for cartilage regeneration should provide not only mechanical support but porous architecture. Pore size itself is also critical. It needs to be large enough to allow cell movement and complete ECM synthesis and small enough to allow cells to attach to a broad surface area (Zhang et al., 2014).

Despite the limits mentioned above, nanocellulose holds promise as a cartilage tissue engineering scaffold. In the long run, this biomaterial has the potential to improve life quality and comfort. However, more research is needed to investigate the long-term impacts of the NC-based scaffolds on the physiology of chondrocyte or stem cells.

## Glossary

ACAN: aggrecan coding gene
a-CNCs: aldehyde-functionalized cellulose nanocrystals
ALP: alkaline phosphatase
ACI: autologous chondrocyte implantation
ACs: articular chondrocytes
ATSCs: stem cells from adipose tissue
BM-MSCs: bone marrow mesenchymal stem cells
BMP: bone morphogenetic protein
BNC: bacterial cellulose
CA: cellulose acetate
CaP: carbonated apatite
CBFB: transcription factor core binding factor beta subunit
CMC: carboxymethylcellulose
CMCA: amidic derivative of CMC
CNC: cellulose nanocrystals
COL1A1: collagen type I α1 chain coding gene
COL2A1: collagen type II α1 chain coding gene
COL10A1: X collagen type X α1 chain coding gene
COMP: cartilage oligomeric matrix protein
CPC: calcium phosphate cement
CS: chitosan
2D: two-dimensional
3D: three-dimensional
Dex: dextran
ECM: extracellular matrix
ESCs: embryonic stem cells
FGF: fibroblast growth factor
FN: fibronectin
GAG: glycosaminoglycan
GelMA: gelatin methacrylate
GP: β-glycerophosphate
HA: hyaluronic acid
HAMA: hyaluronic acid methacrylate
hBM-MSCs: human bone marrow mesenchymal stem cells
hDPSCs: human dental pulp stem and progenitor cells
H&E: hematoxylin and eosin
HEC: hydroxyethylcellulose
hNCs: human nasoseptal chondrocytes
HPC: hydroxypropyl cellulose
HPMC: hydroxypropylmethylcellulose
IFP-MSCs: infrapatellar fat pad derived mesenchymal stem cells
iPSCs: induced pluripotent stem cells
KNG: kartogenin
LiBr: lithium bromide
MACI: matrix-induced autologous chondrocyte implantation
MC: methylcellulose
MMP: matrix metalloproteinase
MRI: magnetic resonance imaging
MSCs: mesenchymal stem cells
NaCS: completely sulfated version of sodium cellulose
NC: nanocellulose osteoarthritis
NFC: nanofibrillated cellulose, OA
PAA: poly(acrylic acid)
PCL: polycaprolactone
PEGDA: polyethylene glycol diacrylate
PLA: poly(d,l-lactide)
P-CNC: phosphated cellulose nanocrystals
pSC: partially sulfated cellulose
PU: polyurethane
RUNX: Runt-related transcription factor
semi-IPNs: semi-interpenetrating polymer networks
sCMC: sulfated carboxymethylcellulose
S-CNC: sulfated cellulose nanocrystals
sGAG: sulfonated glycosaminoglycan
SF: silk fibroin
Si-HPMC: silanized-hydroxypropyl methylcellulose
SOX9: SRY-Box Transcription Factor 9
UC-MSCs: umbilical cord mesenchymal stem cells
TE: tissue engineering
TGF-β: transforming growth factor β
USPIO: ultrasmall superparamagnetic iron oxide
VEGF: vascular endothelial growth factor
